# Comparative genomics of *Campylobacter concisus*: Analysis of clinical strains reveals genome diversity and pathogenic potential

**DOI:** 10.1038/s41426-018-0118-x

**Published:** 2018-06-26

**Authors:** Matthew R. Gemmell, Susan Berry, Indrani Mukhopadhya, Richard Hansen, Hans L. Nielsen, Mona Bajaj-Elliott, Henrik Nielsen, Georgina L. Hold

**Affiliations:** 1 Centre for Genome Enabled Biology and Medicine, School of Medicine, Medical Sciences and Nutrition, University of Aberdeen, Aberdeen,, AB25 2ZD UK; 2 GI Research Group, School of Medicine, Medical Sciences and Nutrition, University of Aberdeen, Aberdeen,, AB25 2ZD UK; 3 Department of Paediatric Gastroenterology, Royal Hospital for Children, Glasgow, G51 4TF UK; 4 Department of Clinical Microbiology, Aalborg University Hospital, DK9100 Aalborg, Denmark; 5 Infection, Immunity, Inflammation Programme, UCL Great Ormond Street Institute of Child Health, 30 Guildford Street, London, WC1N 1EH UK; 6 Department of Infectious Diseases, Aalborg University Hospital, DK9100 Aalborg, Denmark; 7 St George & Sutherland Clinical School, University of New South Wales, Sydney, NSW 2052 Australia

## Abstract

In recent years, an increasing number of *Campylobacter* species have been associated with human gastrointestinal (GI) diseases including gastroenteritis, inflammatory bowel disease, and colorectal cancer. *Campylobacter concisus*, an oral commensal historically linked to gingivitis and periodontitis, has been increasingly detected in the lower GI tract. In the present study, we generated robust genome sequence data from *C. concisus* strains and undertook a comprehensive pangenome assessment to identify *C. concisus* virulence properties and to explain potential adaptations acquired while residing in specific ecological niche(s) of the GI tract. Genomes of 53 new *C. concisus* strains were sequenced, assembled, and annotated including 36 strains from gastroenteritis patients, 13 strains from Crohn’s disease patients and four strains from colitis patients (three collagenous colitis and one lymphocytic colitis). When compared with previous published sequences, strains clustered into two main groups/genomospecies (GS) with phylogenetic clustering explained neither by disease phenotype nor sample location. Paired oral/faecal isolates, from the same patient, indicated that there are few genetic differences between oral and gut isolates which suggests that gut isolates most likely reflect oral strain relocation. Type IV and VI secretion systems genes, genes known to be important for pathogenicity in the *Campylobacter* genus, were present in the genomes assemblies, with 82% containing Type VI secretion system genes. Our findings indicate that *C. concisus* strains are genetically diverse, and the variability in bacterial secretion system content may play an important role in their virulence potential.

## Introduction

*Campylobacter concisus*, an oral commensal bacterial species, has emerged as a potential pathogenic entity in gastrointestinal (GI) diseases. *C. concisus* was first described in relation to humans in 1981, when it was isolated from patients with periodontal lesions and was proposed to contribute to gingivitis and gingival destruction^1^. In recent years, it has been associated with diarrhoeal disease as well as inflammatory bowel disease (IBD), which includes Crohn’s disease (CD) and ulcerative colitis (UC)^2–4^. Since its first isolation, several studies have demonstrated its presence in both healthy and diseased individuals, highlighting the possibility that strains may possess different pathogenic potential^5^. These observations raise the hypothesis that pathogenic potential may differ by anatomical site in the same host^6^, as bacterial adaptation must occur depending upon the ecological niche, leading to varying phenotypic expression and, consequently, different host responses.

Previous in vitro studies have shown that *C. concisus* strains can invade host cells, damage intestinal epithelial barriers, induce proinflammatory cytokine production and form biofilms^3,7,8^. *Campylobacter concisus* strains obtained from patients with chronic intestinal disease have also been shown to possess additional putative virulence factors including exotoxin9/DNAI^9^. Based on their virulence potential and the different phenotypes it was proposed that pathogenic *C. concisus* isolates should be categorised as either (1) adherent and invasive *C. concisus* (AICC) or (2) adherent and toxinogenic *C. concisus* (AToCC), in a classification system which echoes that used for *Escherichia coli*, another intestinal pathogen with highly variable strain-level phenotypes^9,10^.

Recently a comparative genome analysis of 36 *C. concisus* strains was published, which included 27 newly sequenced genomes and nine publicly available genomes^11^. The study identified two novel genomic islands, which contained a number of virulence factors only seen in some enteric strains but not in oral strains, highlighting the need to interrogate a larger number of *C. concisus* isolates, both in terms of disease presentation, but also site of isolation by comparing *C. concisus* isolates from the same individual from different anatomical locations. In addition, no studies have interrogated the plasmid component of the *C. concisus* genome. Identification of new genomic features will provide further insights into the evolution and pathogenic potential of *C. concisus*. We therefore performed comparative genome analysis of 53 *C. concisus* strains from 44 patients, including six patients with *C. concisus* strains isolated from both oral and faecal samples. Their genome and plasmid content were compared with the 36 previously published genomes as well as a more general comparison within the *Campylobacter* family. Our analysis provides insight into the genomic potential within *Campylobacteraceae* thus expanding our understanding of microbial adaptation and diversity within the human GI tract.

The recent focus on microbial community analysis of the human gut microbiome, brought about through innovations in sequencing technology is inherently biased toward abundant bacterial communities and against low-abundance clades, which may still have phenotypic significance in disease. Metagenomic approaches also rely on template datasets to help with downstream analysis. Detailed interrogation of potentially important GI organisms such as *C. concisus* is therefore warranted to complement and enrich metagenomics studies.

## Materials and methods

### Bacterial isolates

All bacterial isolates used in the study were obtained from patient samples. Information on sample types and disease presentation are shown in Table 1. All patients gave informed consent, and ethical approval was obtained from the local ethics committees.

**Table 1:** Characteristics and phenotype data for *Campylobacter concisus* strains used in the study

Strain	Patient	Patient group	Sample type	Diagnosis	Country of origin	Sequencing platform
2009–118452	2009–118452	Adult	Faecal	Crohn’s disease	Denmark	IM
2009–119100	2009–119100	Adult	Faecal	Collagenous colitis	Denmark	IM
2009–129008	2009–129008	Adult	Faecal	Gastroenteritis	Denmark	IM
2009–130586	2009–130586	Adult	Faecal	Gastroenteritis	Denmark	IM
2009–158448	2009–158448	Adult	Faecal	Collagenous colitis	Denmark	IM
2009–173039	2009–173039	Adult	Faecal	Gastroenteritis	Denmark	IM
2009–42653	2009–42653	Adult	Faecal	Gastroenteritis	Denmark	IM
2009–75710	2009–75710	Adult	Faecal	Gastroenteritis	Denmark	IM
2009–75775	2009–75775	Adult	Faecal	Crohn’s disease	Denmark	IM
2009–86120	2009–86120	Adult	Faecal	Bloody diarrhoea	Denmark	IM
2009–91522	2009–91522	Adult	Faecal	Crohn’s disease	Denmark	IM
2010–112100-F	2010–112100	Adult	Faecal	Crohn’s disease	Denmark	IM
2010–112100-O	2010–112100	Adult	Saliva	Crohn’s disease	Denmark	IM
2010–112708	2010–112708	Adult	Faecal	Bloody diarrhoea	Denmark	IM
2010–112758	2010–112758	Adult	Faecal	Gastroenteritis	Denmark	IM
2010–112825	2010–112825	Adult	Faecal	Gastroenteritis	Denmark	IM
2010–113332-F	2010–113332	Adult	Faecal	Gastroenteritis	Denmark	IM
2010–113332-O	2010–113332	Adult	Saliva	Gastroenteritis	Denmark	IM
2010–113862	2010–113862	Adult	Faecal	Gastroenteritis	Denmark	IM
2010–113862-O	2010–113862	Adult	Saliva	Gastroenteritis	Denmark	IM
2010–115605-F	2010–115605	Adult	Faecal	Gastroenteritis	Denmark	IM
2010–115605-O	2010–115605	Adult	Saliva	Gastroenteritis	Denmark	IM
2010–131105	2010–131105	Adult	Faecal	Gastroenteritis	Denmark	IM
2010–16206	2010–16206	Adult	Faecal	Collagenous colitis	Denmark	IM
2010–164712	2010–164712	Adult	Faecal	Bloody diarrhoea	Denmark	IM
2010–1718	2010–1718	Adult	Faecal	Crohn’s disease	Denmark	IM
2010–25654-F	2010–25654	Adult	Faecal	Bloody diarrhoea	Denmark	IM
2010–25654-O	2010–25654	Adult	Saliva	Bloody diarrhoea	Denmark	IM
2010–30795	2010–30795	Adult	Faecal	Gastroenteritis	Denmark	IM
2010–30800	2010–30800	Adult	Faecal	Lymphocytic colitis	Denmark	IM
2010–31374	2010–31374	Adult	Faecal	Bloody diarrhoea	Denmark	IM
2010–33561	2010–33561	Adult	Faecal	Crohn’s disease	Denmark	IM
2010–34330	2010–34330	Adult	Faecal	Crohn’s disease	Denmark	IM
2010–347972	2010–347972	Adult	Faecal	Gastroenteritis	Denmark	IM
2010–36743	2010–36743	Adult	Faecal	Gastroenteritis	Denmark	IM
2010–378007-F	2010–378007	Adult	Faecal	Gastroenteritis	Denmark	IM
2010–378007-O	2010–378007	Adult	Saliva	Gastroenteritis	Denmark	IM
2010–43100	2010–43100	Adult	Faecal	Gastroenteritis	Denmark	IM
2010–6073	2010–6073	Adult	Faecal	Gastroenteritis	Denmark	IM
2010–8194	2010–8194	Adult	Faecal	Gastroenteritis	Denmark	IM
2010–88823	2010–88823	Adult	Faecal	Gastroenteritis	Denmark	IM
2012–164712	2012–164712	Adult	Faecal	Bloody diarrhoea	Denmark	IM
2012–191940	2012–191940	Adult	Faecal	Gastroenteritis	Denmark	IM
2012–37302	2012–37302	Adult	Faecal	Gastroenteritis	Denmark	IM
2013–101463	2013–101463	Adult	Faecal	Gastroenteritis	Denmark	IM
2013–39845	2013–39845	Adult	Faecal	Gastroenteritis	Denmark	IM
2013–42088	2013–42088	Adult	Faecal	Gastroenteritis	Denmark	IM
2013–87946	2013–87946	Adult	Faecal	Gastroenteritis	Denmark	IM
B124_Slimy-small	B124	Paediatric	Biopsy	Crohn’s disease	UK	IM
B124_Small-clear	B124	Paediatric	Biopsy	Crohn’s disease	UK	IM + PB-RSII
B124_Small-grey	B124	Paediatric	Biopsy	Crohn’s disease	UK	IM
B38_Tiny-mucoid	B38	Paediatric	Biopsy	Crohn’s disease	UK	IM + PB-RSII
B124_Slimy-large	B124	Paediatric	Biopsy	Crohn’s disease	UK	IM

*IM* Illumina MiSeq, *PB-RSII* PacBio-RSII

### Ethics approval and consent to participate

Scientific and ethics approval for the study was obtained from the Ethics Committee for North Denmark Region (N-20080056 and N-20110008) and North of Scotland Research Ethics Service (09/S0802/24 and 12/NS/0061).

### Public data acquisition

Assembled genomes and read data described in Deshpande et al.^41^ were acquired in July 2016 and all subsequent analysis carried out on the downloaded datasets. Raw read data from Chung et al.^11^ was acquired in March 2017. These were under BioProject number PRJNA348396 in the GenBank Sequence Reads Archive. The raw reads form Chung et al.^11^ were assembled as described in the Methods section below and further analysis carried out on the assembled genomes.

### Genome sequencing

We performed short-read sequencing for all 53 strains, of which two (B38_Tiny mucoid and B124_Small-clear) were also subjected to long-read sequencing. For short-read sequencing, genomic DNA was extracted from *C. concisus* liquid cultures using the Promega Genomic DNA Purification kit. Library construction was performed for all strains using the Illumina Nextera XT DNA Library Prep Kit and Nextera XT Index Kit v2, quantified by the 2100 Bioanalyzer (Agilent Technologies), and then sequenced on the Illumina MiSeq with MiSeq Reagent Kit v3 (Illumina, Inc) for 600 cycles and 2 × 300 bp paired end reads. The quality of raw paired-end reads was checked using FastQC (http://www.bioinformatics.babraham.ac.uk/projects/fastqc/). For long-read sequencing, genomic DNA was extracted using the Qiagen MagAttract High Molecular Weight DNA kit. Samples were sequenced on single-molecule, real time (SMRT) cells using Pacific Biosciences RS II (Pacific Biosciences, Menlo Park, CA) at the University of Liverpool Centre for Genomic Research (CGR).

### PCR validation of Zot and Exotoxin 9 genes

The Zot PCR was performed using the primer pair Zot1 (GCAACTTAGAAAAAGTATCGG) and Zot2 (TAATAGTTCTCGATGAAGCC), which amplifies a 979 bp region, and the primer pair ZotF (CTAGAATCAGTTTGTGGAGAT) and Zot2, which amplifies a 790 bp region as previously described^12^. Exotoxin 9 DNA was amplified using the following primers: 5′-GAGACAAAGCTGCTTTAT-3′ (exotox-F) and 5′-CTATCAAGATTAAAGCCG-3′ (exotox-R) as previously described^13^.

### De novo assembly and gene annotation

Strains with only short-read sequencing were error corrected and assembled with the default settings of the A5-miseq pipeline^14^. Strains with long-read sequencing (B38_Tiny-Mucoid and B124_Small-clear) were error corrected and assembled with the hierarchical genome-assembly process (HGAP3)^15^.

To generate more complete genome assemblies for the strains with only short-read sequencing we used SSPACE-LongRead to scaffold short-read assemblies with PacBio filtered subreads (provided from CGR). All A5-miseq assemblies were scaffolded with B38_Tiny-mucoid. The exception for this was B124_Small-clear which was scaffolded with B124_Small-clear. During analysis, many assemblies were created for each sample and compared with the best assembly chosen for downstream analysis and submission. After SSPACE-LongRead assembly, the final scaffolds for each strain were gap filled with GapFiller^16^ using the error corrected paired Illumina MiSeq reads (error correction carried out by A5-miseq pipeline step 1) from its respective sample.

Genome assemblies were annotated with Prokka^17^ along with the recommended and optional tools Aragorn^18^, Barrnap (https://github.com/Victorian-Bioinformatics-Consortium/barrnap), HMMER3^19^, Infernal^20^, RNAmmer^21^, and SignalP^22^. Quality Assessment Tool for Genome Assemblies (QUAST) was used to produce genome assembly evaluation metrics^23^. BUSCO v2 was used to detect Bacterial Benchmarking Universal Single-Copy Orthologs (BUSCOs) in the amino acid sequence fasta files created by Prokka to assess genome completeness^24^. The errors within the genome assemblies of this study were evaluated by Recognition of Errors in Assemblies using Paired Reads (REAPR)^25^. Prior to REAPR, reads were preprocessed with BWA^26^, The FastX toolkit (http://hannonlab.cshl.edu/fastx_toolkit/) and cmpfastq (http://compbio.brc.iop.kcl.ac.uk/software/cmpfastq.php). Blobtools was utilised to detect and filter out contamination from some of the genome assemblies^27^.

We assembled plasmids for strains in the current study and genomes from publicly available databases (NCBI and DDBJ) using plasmidSPAdes^28^. The algorithm uses the whole genome sequencing reads to assemble plasmids and removes chromosomal contigs from the plasmid assembly. Plasmid assemblies were annotated with Prokka^17^. QUAST was used to produce plasmid assembly contig metrics^23^.

### Comparative genomics approaches

A total of 23S rRNA sequences were extracted from the genome assemblies using Barrnap (http://www.vicbioinformatics.com/software.prokka.shtml), this produced a .gff of rRNA locations in the genome assemblies. The .gff files were used in combination with the bedtools^29^ command, fastaFromBed, to extract the 23S rRNA sequence from the genome assemblies. Phylogenetic tree based on 23S rRNA was produced using MEGA6^30^. DNA sequences were aligned using the MUSCLE^31^ alignment algorithm. MUSCLE settings were, gap open-400, gap extend 0, clustering method (Iteration 1.2) UPGMB, clustering method (Other Iterations) UPGMB, and min diag length (lambda) 24. Phylogeny reconstruction was carried out with the statistical method of neighbour-joining, test of phylogeny set to bootstrap method with 1000 replications, Substitutions type set to nucleotide with a *p*-distance model and substitutions to include being transitions and transversions, Rates among sites was set to uniform, pattern among lineages set to same (homogeneous), and gaps/missing data treatment set to complete deletion.

A phylogenetic tree based on the full genome content of the assembled genomes of the *C. concisus* strains was produced using reference alignment based phylogenetic builder (REALPHY) and RAxML^32,33^. REALPHY was run using the genbank files of the genomes (.gbk) created by Prokka annotation. REALPHY defaults were used with BOWTIE2, BOWTIE2BUILDER, Rscript, and RAxML executables locations included in the config.txt file^33,34^. RAxML was run on the “polymorphisms_move.phy” and “model.txt” files produced by REALPHY, which contain the alignment data file in PHYLIP format (-s) and the file which contains the assignment of models to alignment partitions for multiple models of substitution (-q), respectively. Phylogeny was estimated with the generalised time reversible (GTR) nucleotide substitution model and optimisation of substitution rates and GAMMA model of rate heterogeneity (RAxML flags-m GTRGAMMA-p 749889). RAxML was carried out with rapid bootstrap analysis and a search for the best-scoring ML tree with 1000 replications (RAxML flags -f a -x 12345 -N 1000). The type strain *Campylobacter curvus* 525.92 (NCBI Reference Sequence: GCF_000017465.2) was used as an outgroup for the phylogenetic analysis.

The Comprehensive Antibiotic Resistance Database (CARD) and Virulence Factor Database (VFDB) were used to detect antibiotic resistance genes and virulence factors in annotated genes from assembled genomes and plasmids (Both downloaded September 2016)^35,36^. The *C. concisus* genome and plasmid assemblies were compared with CARD with the Resistance Gene Identifier (RGI) (Version 3.1.1), including loose hits. Blastn was used to align the VFDB nucleotide core and full dataset, acting as the target, against the genome and plasmid assemblies, acting as the query, with an *e*-value threshold of 1e-04.

### Pangenome analysis

Pangenomes were deduced using the pan genome pipeline Roary^37^ using gff files produced by Prokka. Roary was carried out using the -e (create a multiFAST alignment of core genes using PRANK), -n (fast core gene alignment with MAFFT, and the -v (verbose output to STDOUT) parameters. Two sets of pangenomes were created. The first set contained all the *C. concisus* genomes whilst the second contained all the *C. concisus* and one reference genome assembly for 20 other *Campylobacter* species. These other *Campylobacter* genome assemblies were *Campylobacter showae* ATCC51146, *Campylobacter jejuni* NCTC 11168, *Campylobacter coli* OR12 (NCBI Reference Sequence: NZ_CP013733.1), *Campylobacter fetus subsp. testudinum* 03-427, *Campylobacter lari* RM2100, *Campylobacter ureolyticus* RIGS 9880, *Campylobacter upsaliensis* JV21 (NCBI Reference Sequence: NZ_AEPU00000000.1), *C. curvus* 525.92 (NCBI Reference Sequence: NC_009715.2), *Campylobacter subantarcticus* LMG 24377 (NCBI Reference Sequence: NZ_CP007773.1), *Campylobacter gracilis* strain ATCC 33236, *Campylobacter rectus* RM3267, *Campylobacter hominis* ATCC BAA-381 (NCBI Reference Sequence: NC_009714.1), *Campylobacter volucris* LMG 24379 (NCBI Reference Sequence: NZ_CP007774.1), *Campylobacter sputorum* INTA08/209, *Campylobacter cuniculorum* DSM 23162 (NCBI Reference Sequence: NZ_JHZL00000000.1), *Campylobacter insulaenigrae* NCTC 12927, *Campylobacter hepaticus* strain HV10 (NCBI Reference Sequence: NZ_LUKK00000000.1), *Campylobacter corcagiensis* strain CIT 045, *Campylobacter iguaniorum* strain 1485E (NCBI Reference Sequence: NZ_CP009043.1) and *Campylobacter mucosalis* strain DSM 21682 (NCBI Reference Sequence: NZ_JHQQ00000000.1).

Roary produces a fasta file which contains a representative sequence for each gene in the inferred pangenome. These genes were classified by KEGG Orthology (ko0001) KEGG BRITE hierarchies. Prior to classification the gene sequences within the Roary produced representative gene fasta file were translated into amino acids with the EMBOSS command, transeq. Translated amino acid sequences were classified by BlastKOALA^38^.

### Plasmid comparison

The amino acid sequence files (.faa) produced by Prokka^17^ from the plasmid contig files were annotated with BlastKOALA^38^. This was carried out to annotate the amino acid sequences with KEGG Orthology (ko0001) KEGG BRITE hierarchies. This allowed for investigation of the contents of the plasmids in each sample.

### Figure creation

Phylogenetic tree visualisations were produced using the online software iTOL, v3.5.4^39^. Colouring was chosen using ColorBrewer (Brewer, Cynthia A., 200×. http://www.ColorBrewer.org, accessed 8 Dec 2016).

Various figures were created using the Statistical Language and Environment R (Figs. 2–6b, Supplementary Figure 1, Supplementary Figure 3, Supplementary Figure 4, and Supplementary Figure 5). For full annotated scripts see Additional File 3 (http://www.R-project.org/). The R package, ggplot2, was used to create barcharts and poly frequency plots (Fig. 4a, b, Supplementary Figure 1, Supplementary Figure 3, Supplementary Figure 4 and Supplementary Figure 5). The R package gplots was used to create hierarchically clustered heat maps (Figs. 2, 3, 5, and 6b) (https://CRAN.R-project.org/package = gplots). The package RColorBrewer was used to choose colours for grouping in the R plots. RColorBrewer: ColorBrewer Palettes. R package version 1.1–2. https://CRAN.R-project.org/package = RColorBrewer). R packages reshape, reshape2 and tidyr were utilised to manipulate data with the R environment (URL http://www.jstatsoft.org/v21/i12/; https://CRAN.R-project.org/package = tidyr). The R package knitr was used to produce the R markdown in Additional File 3.f.

## Results

### Sequencing and assembly of *Campylobacter concisus* genomes, contiguity, completeness, and accuracy assessment

Fifty-three clinical *C. concisus* strains were obtained from 44 patients (42 adult and two paediatric; Table 1). Thirty-six isolates were from patients with gastroenteritis, and for five patients’ strains were available from the oral cavity as well as from faecal samples. Thirteen *C. concisus* strains were isolated from CD patients: seven isolates from faecal samples; for one patient strains were available from both oral and faecal samples. A further five isolates were obtained from paediatric Crohn’s patient biopsies from inflamed areas of the colon at initial diagnosis. A further four strains (2009-119100, 2009-158448, 2010-16206, and 2010-30800) were isolated from faecal samples from microscopic colitis patients (three collagenous colitis and one lymphocytic colitis).

The genome sizes of the isolates ranged from 1.85 to 2.29 Mb, with the exception of two strains – 2010-115605-O and 2010-347972 which had larger than expected genome assembly sizes. Due to large genome assembly sizes, the presence of contamination was tested for in these samples. Contamination was detected in both but was only successfully removed in the genome assembly of 2010-347972. Due to this 2010-115605-O was excluded from further downstream analysis.

The fold coverage for Illumina sequencing ranged from 32 to 114 (Supplementary table 1). Pacbio sequencing fold coverage was 175 and 696 for B124_Slimy_small and B38_Tiny_mucoid respectively. On average the new genomes contained 1928 Coding DNA Sequences (CDS) within the range of 1769–2075. There was an average of 2006 unique genes within the range of 1811–2115 genes (Supplementary table 2). The longest contig from the current study of genomes made up, on average, greater than 32% of their total genome length (Supplementary Figure 1). The vast majority of the new genomes were estimated to be 97% or more complete by BUSCO analysis (Supplementary Figure 1; Supplementary table 3).

The accuracy of the genomes from this study were assessed by REAPR. This found that the assemblies for thirteen of the isolates had very low amounts of errors with >90% of assemblies being error free (Supplementary Figure 1; Supplementary table 4). Greater than 80% of the assemblies for a further 33 isolates were error free, indicating low error.

### Phylogenetic relationship of *C. concisus* strains by whole genome sequencing

**Fig. 1:**
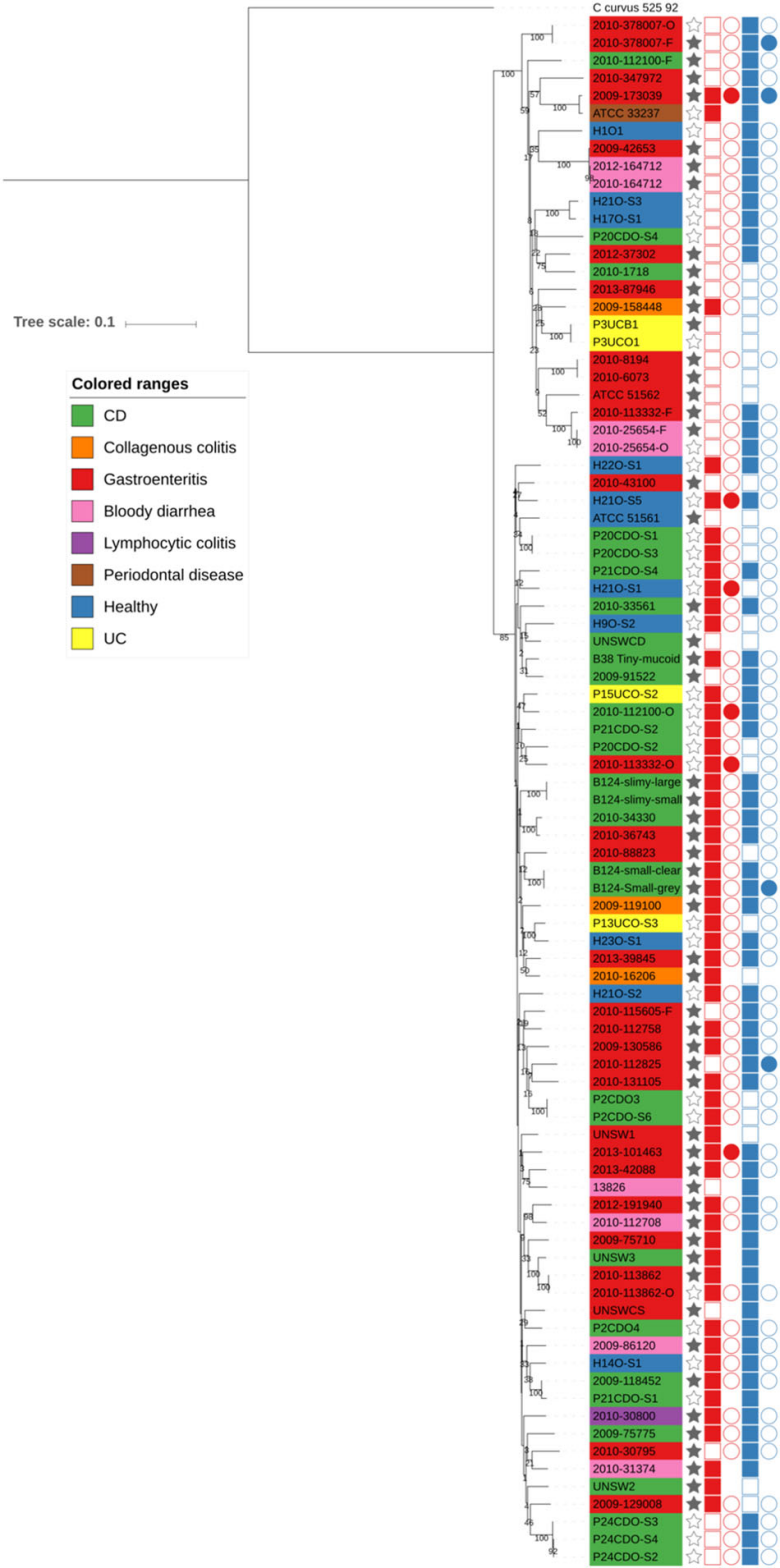
Phylogenetic tree based on whole genome sequences of *Campylobacter concisus* strains used in this study, incorporating all published genomes. Columns: Full stars represent Faecal samples, empty stars represent Oral samples. Squares represent presences in genome assemblies and circles presence in plasmid assemblies. Red represents Exo9 whilst blue represents ZOT presence. Full shapes indicate presence, empty shapes indicate absence. For plasmids, there are some samples with no shape, this indicates that plasmidSPAdes software was unable to assemble any plasmids for the sample

We next compared the 52 new draught genomes with all publicly available *C. concisus* genomes, including the recently deposited 36 *C. concisus* genomes isolated from IBD patient saliva and intestinal biopsy samples^11^, drawing together genome data on 88 distinct *C. concisus* isolates. Previous studies have shown that *C. concisus* has two genomospecies (GS) which potentially differ in pathogenic potential^40^. We incorporated all publicly available genomes into our analysis and initially investigated their phylogenetic relatedness based on 23S rRNA sequences. This divided the genomes into two clusters, with 49 strains clustering together while the remaining 39 strains belonged to a second cluster (Supplementary Figure 2). Both clusters contained both oral and enteric strains, but the clustering did not match the previously published GS clustering, with some reference genomes switching clusters between our study and published findings^11^. Previous data had demonstrated that analysis based on 16S rRNA gene sequences was not effective at differentiating *C. concisus* species^41^. Based on our findings, clustering on 23S rRNA sequences, after inclusion of 53 additional genome sequences, was also not effective. To overcome reproducibility issues with the 23S rRNA phylogenetic analysis, we carried out whole genome phylogenetic analysis (Fig. 1). The whole genome analysis divided the 88 *C. concisus* strains into GSI and GSII encompassing 25 and 63 strains, respectively, with segregation of the published genomes as previously described^11^. Bootstrap values were high indicating the inferred phylogenetic tree was highly reliable. When the origin of the *C. concisus* isolates was assessed based on GS, it was apparent that isolates collected from healthy individuals were present in both groups whilst isolated obtained from CD patients were mainly clustering within GSII.

### Factors mediating *Campylobacter concisus* pathogenicity and its interaction with the host environment

We looked for the presence of virulence factors within the genomes, focussing initially on assessing zonula occludens toxin (zot) and exotoxin 9 as these have been previously reported in *C. concisus*^13,42^. Zot positivity was identified in 72% of genomes, with presence/absence further validated by direct PCR in the 53 genomes of this study. Sixty-eight percent of GSI strains were Zot positive compared with 73% of GSII strains (Fig. 1; chi-square statistic = 2.235, *p* = 0.135). The rate of Zot positivity was slightly higher than for exotoxin 9, with 62% of all genomes confirmed as containing the exotoxin 9 gene sequence. Again presence/absence was confirmed experimentally by PCR in the new 53 isolates. Only 12% of GSI strains were exotoxin 9 positive, however, compared with 81% of GSII strains (Fig. 1; chi-square statistic = 35.89, *p* < 0.00001). Forty-two percent of strains contained both putative virulence factors with only 11% of strains containing neither. When ZOT and exotoxin 9 positivity were compared based on the disease status of the patients from which strains were isolated, isolates obtained from CD patients demonstrated the highest level of positivity whilst isolates obtained from patients with collagenous colitis and UC demonstrated the lowest rates of positivity although this could be due to small numbers of isolates from these patient groups. The rates of positivity, however, for strains obtained from healthy patients was only slightly lower than the CD patients but exotoxin 9 positivity was higher in healthy subjects than gastroenteritis patients (Table 2).

**Table 2:** Clinical source of strains based on ZOT and Exotoxin 9 positivity as well as the percentage of isolates that belonged to Genomospecies (GS) I and II

Clinical source	Total strains	ZOT positivity	Exotoxin 9 positivity	GSI	GSII
CD	29	72.41	72.41	10.34	89.66
Gastroenteritis	31	70.97	51.61	35.48	64.52
Lymphocytic colitis	1	100	100	0	100
Collagenous colitis	3	33.33	100	33.33	66.67
Bloody diarrhoea	8	100	37.5	50	50
Periodontal disease	1	100	100	100	0
Healthy	11	72.73	63.64	27.27	72.73
UC	4	25	50	50	50
All	88	71.59	60.22	28.41	71.59

*GS* genomospecies; *CD* Crohn’s disease, *UC* ulcerative colitis

To identify other genes that may contribute to virulence, we aligned genes and proteins from each strain to the Comprehensive Antibiotic Resistance Database (CARD) and Virulence Factor Database (VFDB). The VFDB contains a core dataset (“core” VFDB) which contains only experimentally verified VFs (virulence factors) and another full dataset (“all” VFDB) containing all known and predicted VFs.

**Fig. 2:**
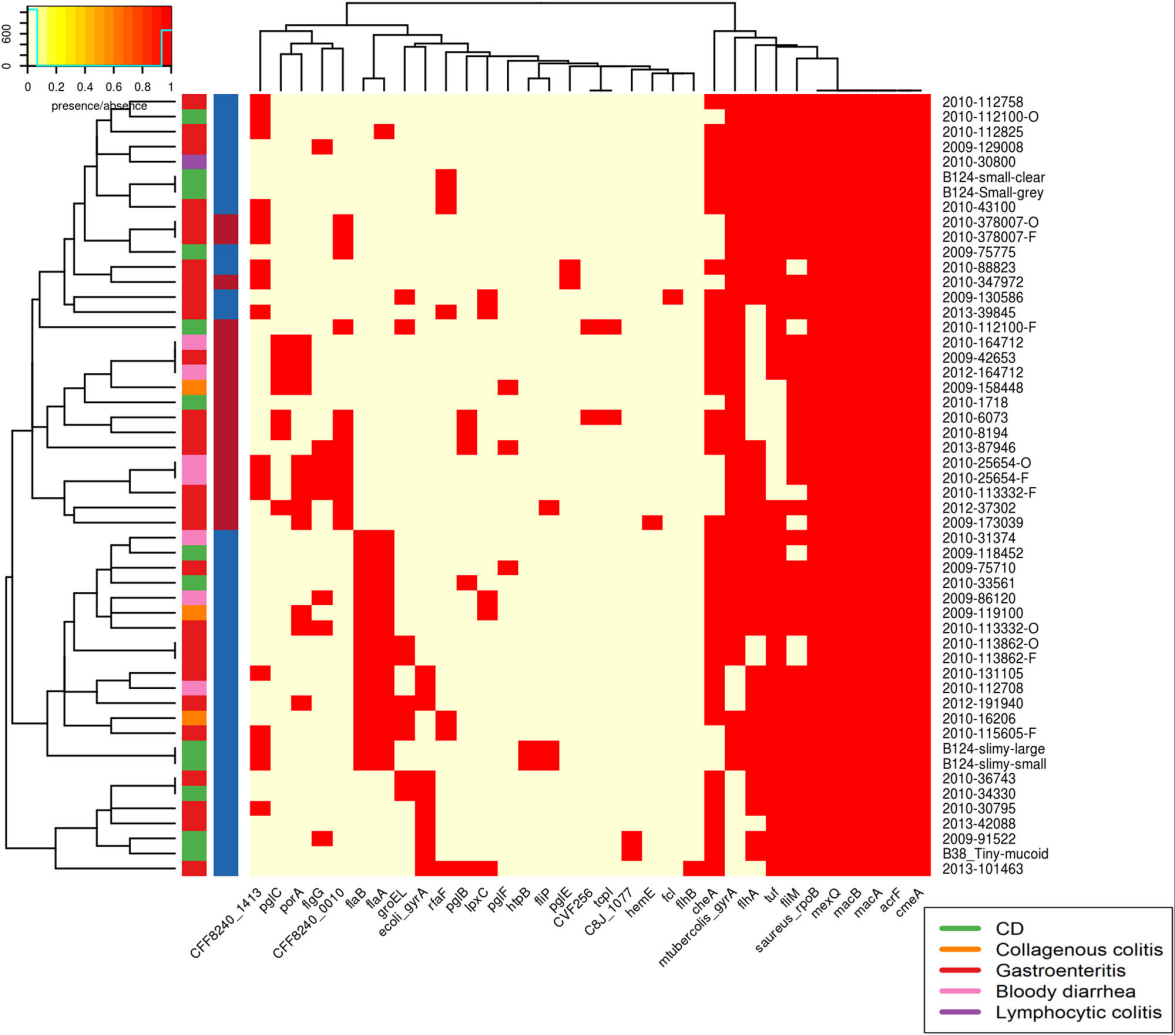
Presence of virulence factors in genomes of isolates sequenced for this study. Contains VFDB hits from the VFDB all dataset. Virulence factors were detected using the Comprehensive Antibiotic Resistance Database (CARD) and the Virulence Factor Database (VFDB). There are two colour columns representing metadata for the samples. The first represents the disease presentation of the host with a legend available. The second represent the genomospecies (GS) of the isolate with “red” referring to GSI and “blue” to GSII

The *C. concisus* genomes aligned poorly to both reference databases, indicating that *C. concisus* genes are only distantly related to well-characterised pathogens. Most of the isolates showed the presence of seven or more antibiotic resistance genes from CARD, whilst most isolates contained <6 VFs, from the core VFDB set, with a maximum of 14 (Supplementary table 5). This value differs greatly to that seen in *C. jejuni*, with 160 and 636 VFs identified, using VFDB analysis with the core VFDB, and all VFDB for *C. jejuni* NCTC 11168^43^. Due to the relatively uncharacterised nature of *C. concisus* the more comprehensive “all VFDB”dataset was used. When blastn was carried out against the full VFDB dataset, the isolates contained 3–72 VFs. Of the antibiotic resistance genes and VFs, six were present in all samples. These were saureus_rpoB, mexQ, macB, macA, acrF, and cmeA (Fig. 2). All six factors were identified as proteobacterial genes involved in active transport processes, with the exception of saureus_rpoB which is involved in DNA binding but has previously been linked with antibiotic resistance in *Staphylococcus aureus*^44^.

**Fig. 3:**
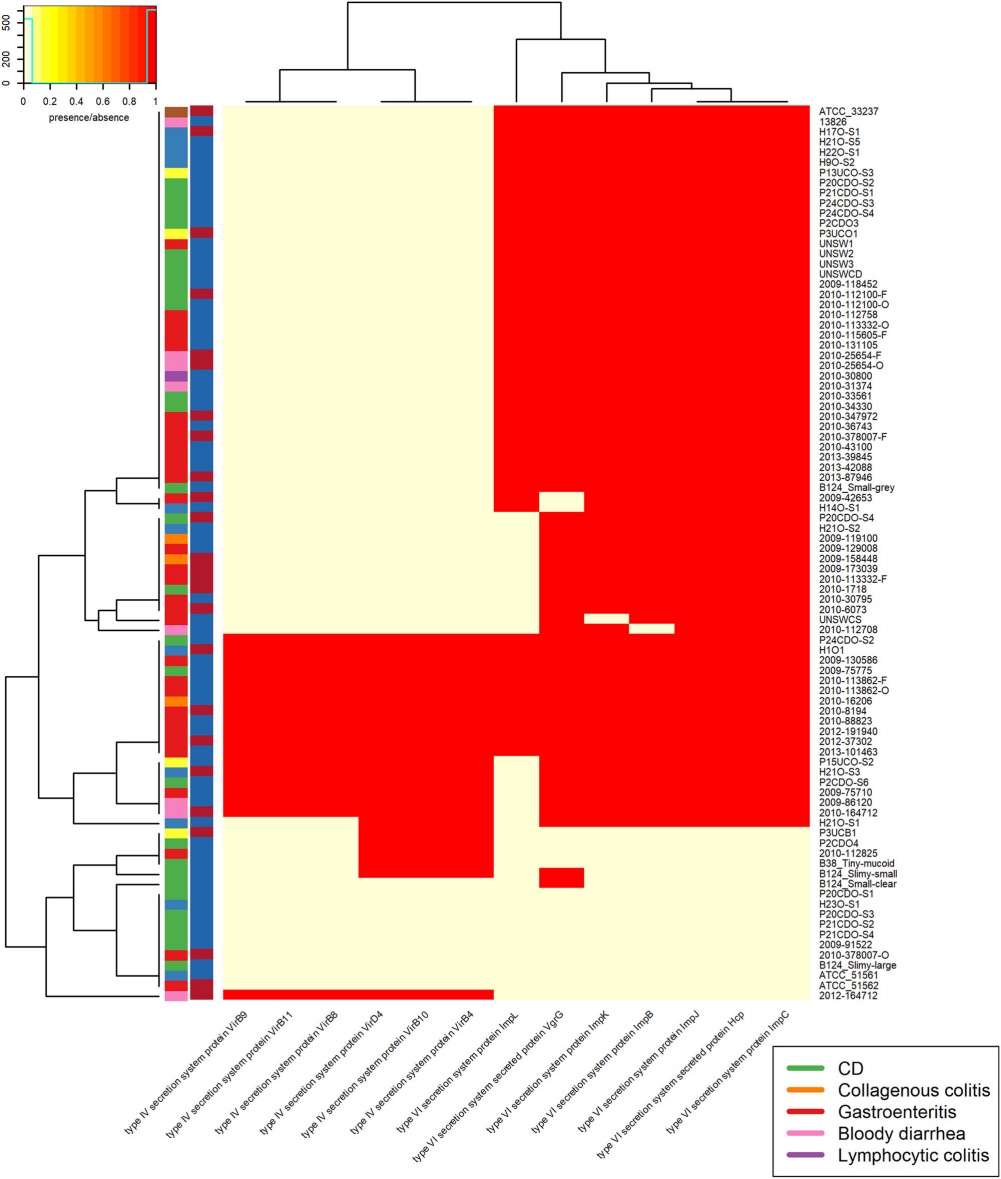
T4SS and T6SS presence within the assembled genomes. There are two colour columns representing metadata for the samples. The first represents the host disease presentation with a legend available. The second represent the GS of the isolate with red referring to GSI and blue to GSII

We also looked for the presence of bacterial secretion systems within the genomes, focussing specifically on the Type IV (T4SS) and Type VI (T6SS) secretion systems. Very few of the *C. concisus* isolates had evidence of a T4SS within their genomes (Fig. 3). In contrast, the presence of a T6SS system was more ubiquitous with 83% of *C. concisus* genomes having T6SS genes present.

### Pangenome analysis of *Campylobacter concisus*

We next generated the *C. concisus* pangenome, combining the genomes of this study and all publicly available *C. concisus* genomes, to identify genetic and functional elements that are shared or distinct among strains. Pangenome analysis defined a total of 14,527 genes of which there were 541 core genes (present in 99–100% of genomes), 97 soft core genes (present in 95–99% of genomes), 2313 shell genes (present in 15–95% of genomes) and 11,576 cloud genes (present in 0–15% of genomes) indicating that ~30% of a single isolate’s genes (based on average gene amount of 2000) are core or soft-core genes and that there are many genes present as cloud genes (Supplementary table 6). As expected, many of the core genes were involved in metabolism, genetic information processing, environmental information processing and cellular processes (Supplementary Figure 3). In terms of functionality, genes linked to human diseases including cancers, cardiovascular diseases, drug resistance, endocrine and metabolic diseases, infectious diseases, and neurodegenerative diseases were found within the core, shell, and cloud gene lists. Human disease-related genes found in all isolates were pyruvate kinase, argininosuccinate synthase, thioredoxin 1, penicillin-binding protein 1A, 2, and 3, chaperonin GroEL (penicillin-binding protein 3), beta-N-acetylhexosaminidase, UDP-N-acetylglucosamine acyltransferase UDP-N-acetylmuramoyl-tripeptide–D-alanyl-D-alanine ligase, ubiquinol-cytochrome c reductase iron-sulphur subunit, ubiquinol-cytochrome c reductase cytochrome b subunit, putative protease, molecular chaperone DnaK, glyceraldehyde 3-phosphate dehydrogenase, N-acetylmuramoyl-L-alanine amidase, glucosamine–fructose-6-phosphate aminotransferase (isomerising), glycine hydroxymethyltransferase, GTP-binding protein LepA, N-acetylmuramoyl-L-alanine amidase, phosphoribosylaminoimidazolecarboxamide formyltransferase, cyclohydrolase, and superoxide dismutase, Cu-Zn family.

**Fig. 4:**
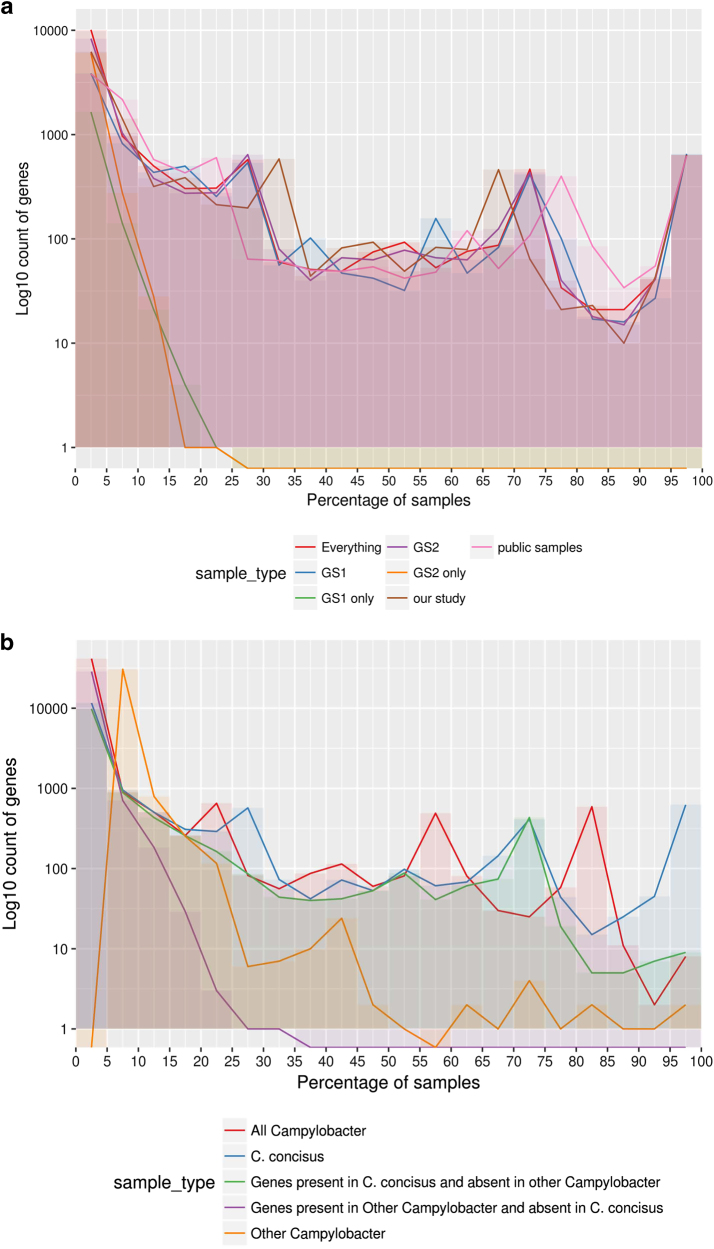
**a** Pangenome summary of all *C. concisus* used in this study. The figure displays the number of genes found to be shared across a certain percentage of samples in different groupings of isolates. This figure uses a bin size of 5% on the *x*-axis. GS Genomospecies, and **b** Pangenome summary of all *Campylobacter*. This was produced by carrying out pangenome analysis of all the *C. concisus* isolates and one reference assembly for each non-*C*. *concisus* species that was available. The figure displays how many genes were found to be shared across a certain percentage of samples in different groupings of isolates. The figure depicts a bin size of 5% on the *x*-axis

A comparison was undertaken to see if pangenome analysis revealed GSs centric features. When comparing GSI and GSII, there was very little difference in core gene content with no core or soft-core genes unique to either GS (Supplementary table 6). There were five shell genes unique to GSI and two to GSII. Genes unique to GSI and GSII were shared across <30% of the GSI and GSII isolates respectively (Fig. 4a). Interestingly only 1815 genes were unique to GSI, but a larger number – 6320 gene – were unique to GSII isolates. The difference in uniques genes between is most likely due to the difference in the number of isolates with an approximate ratio of 24:10 for isolates and a ratio of unique genes of 35:10 when comparing GSII to GSI.

### Genome comparison of *Campylobacter concisus* against other *Campylobacter* species

Further pangenome analysis was carried out to allow a comparison between *C. concisus* against the reference genomes of other *Campylobacter* species. Three core genes and five soft core genes were found across all the *Campylobacter* isolates from a total set of 45,604 genes (Supplementary table 7, Fig. 4b). A total of 624 genes were defined as core or soft-core genes for *C. concisus*, when a comparison was made across all *C. concisus* genomes, whilst only two core or soft-core genes were present across the other *Campylobacte*r species once *C. concisus* was removed. Interestingly, there was little overlap of the genes between the *C. concisus* isolates and other *Campylobacter*. Only 11% of the genes from other *Campylobacter* were found within the *C. concisus* pangenome and only 14% of *C. concisus* genes were found within the other *Campylobacter* genomes. The functionality of the pangenome (Supplementary Figure 4b) of *Campylobacter* indicated there is no major difference at high KEGG BRITE hierarchies between *C. concisus* and other *Campylobacter* species.

### Plasmid analysis

We next compared plasmids across the strains where we had access to the raw sequencing reads. We were able to do this for 78 isolates, with plasmids identified and assembled for 70 isolates. Due to contamination in sequencing reads 2010-347972 was excluded from plasmid assembly and analysis. The plasmid content assembled varied greatly, with an average of 69 kbp total of plasmids across an average of 12 contigs (Supplementary table 8). The average number of genes across the assembled plasmid content was 67 (Supplementary table 9).

In order to account for the possibility of non-plasmids being assembled, downstream analysis looked at the functionality of plasmid genes. Using the CARD and VFDB databases, we found very few VFs. No hits were detected with CARD. No VFs were detected using the VFDB core set, and only five hits using the “all” VFDB set: 1 hit occurred for 2010-11285, 2 for 2010-115605-O, and 2 for 2010-378007-O.

**Fig. 5:**
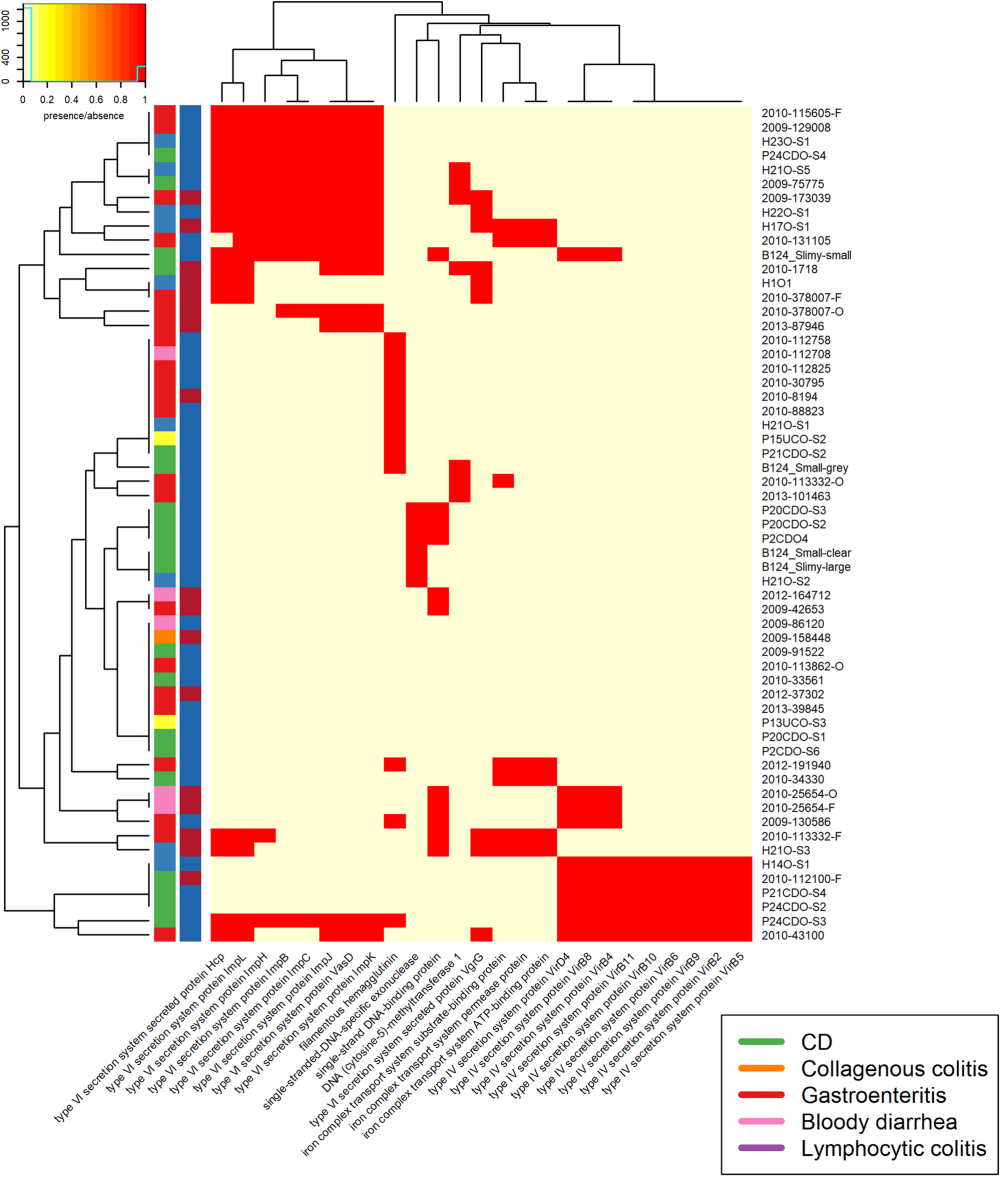
Plasmid KEGG Orthology (ko0001) KEGG BRITE hierarchies present within the amino acid sequences present in plasmid sequences of samples. Hierarchies which were shared by five or fewer isolates were not included. There are two colour columns representing metadata for the samples. The first represents host disease presentation with a legend available. The second represent the GS of the isolate with “red” referring to GSI and “blue” to GSII

Overall there was very little shared functionality of plasmid genes across multiple samples (Supplementary table 10). A total of 30 functional categories were detected in plasmids of 6 or more samples. The majority of these genes were involved in “environmental information and processing” (*n* = 17) and “Cellular processes” (*n* = 6). Looking at these specific genes, there was no clear clustering based on GS or disease presentation (Fig. 5). There was a cluster of Type IV secretion system proteins (VirD4, VirB2, VirB4-6, and VirB8-11), which were found in the assembled plasmids of six isolates. There was also a cluster of Type VI secretion system proteins (Hcp, ImpL, ImpH, ImpB, ImpC, ImpJ, VasD, and ImpK) which were all found in the plasmids of ten isolates.

### Comparison of paired *Campylobacter concisus* isolates from the same patient

**Fig. 6:**
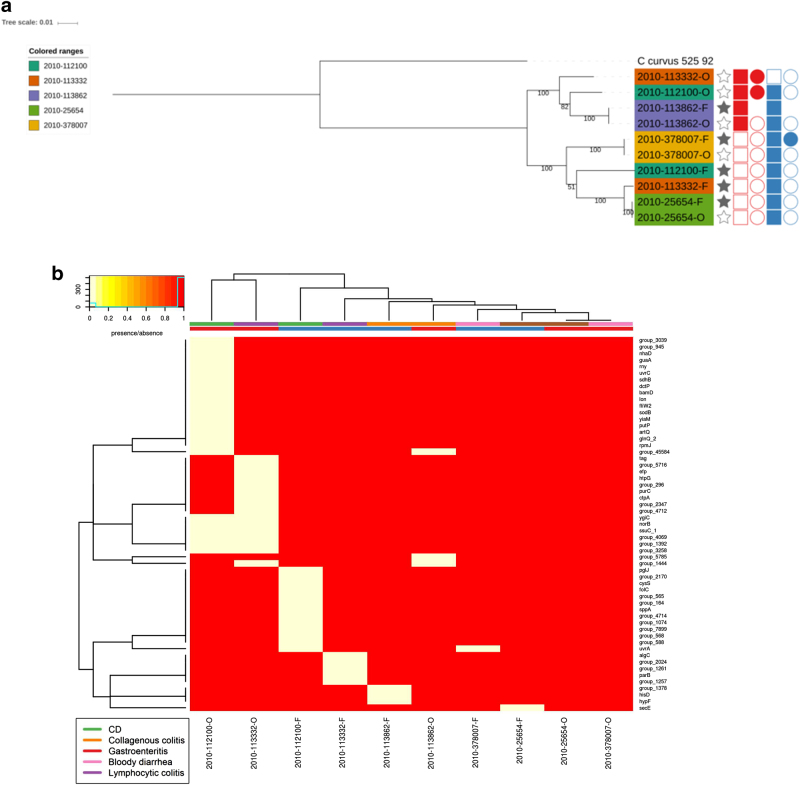
Phylogenetic tree. a Phylogenetic tree of faecal/oral paired samples based on the whole genome and **b** Heatmap of genes found to be core only in oral samples and core in only faecal samples from oral/faecal paired samples. Columns: Full stars represent faecal samples, empty stars represent oral samples. Squares represent presence in genome assemblies and circles presence in plasmid assemblies. “Red” represents Exo9 whilst “blue” represents ZOT presence. Full shapes indicate presence, empty shapes indicate absence. For plasmids, there are some samples with no shape. This indicates that plasmids were not assembled for this sample. Heatmap: There are two colour rows representing metadata for the samples. The first represents the host disease presentation with a legend available. The second represent the GS of the isolate with “red” referring to GSI and “blue” to GSII

Within our *C. concisus* collection, there were five patients for whom *C. concisus* isolate genome sequences were available from both oral and faecal samples. Phylogenetic analysis demonstrated that the isolates did not all cluster by patient or by location (Fig. 6a). The phylogenetic clustering formed two main clusters which matched GS clustering described earlier. Isolates from three patients clustered closely together (2010-113862, 2010-378007 and 2010-25654), although the two paired isolates that did not form a patient cluster were split between the two GS groups (Fig. 6a). This was not by location as in one cluster there were two faecal isolates and in the other, the corresponding oral isolate. Looking at genes that were core only in oral isolates and core only in faecal isolates, within the paired isolates, found that all genes were missing in one or two of the matching faecal/oral isolates (Fig. 6b), thus highlighting the fact that there appears to be no major difference between oral and faecal isolates. These results were reflected upon comparing the pangenomes of the faecal and oral isolates from the five patients. Of the 4859 genes present in the paired isolates, 979 genes were found only in oral isolates whilst 552 genes were found only in faecal strains (Supplementary Table 11). Looking at the number of genes shared across samples, the pattern was very similar for the faecal and oral isolates with the majority of differences seen within shell genes (Supplementary Figure 5).

## Discussion

We conducted a comprehensive comparative genomic analysis of 88 *C. concisus* strains including, for the first time, investigation of their plasmid content. Whole genome sequencing was performed on 53 new *C. concisus* isolates using Illumina short-read sequencing, while two strains were additionally subjected to PacBio long-read sequencing. Our genomes are good quality draught genomes and significantly add to the current knowledge base. The majority of the new genomes were shown to have high contiguity, high completeness, and a low amount of errors. Almost all of the new genomes have 50 or less contigs, whilst the majority of current published genomes have >120 contigs^11^. None of the current study genomes were sequenced to completeness, even following PacBio long read sequencing with B38_Tiny-mucoid and B124_Small-clear comprising 5 and 18 contigs, respectively. Currently only two single contig *C. concisus* genomes are publicly available – 13826 and ATCC 33237^45^.

Our initial attempts to assess *C. concisus* intra-species differences were based on 23S gene sequences, as published data indicated 16S gene sequences did not contain sufficient information to effectively discriminate between strains^41^. Our findings demonstrate however that 23S sequences are also ineffective, with the most likely explanation being the increased burden of additional sequences. A previous study assessing the suitability of 16S rRNA, 23S rRNA and the internal transcribed spacer region (ITS) (between 16S rRNA and 23S rRNA) in the *Campylobacter* genus found that 16S and 23S rRNA are unable to differentiate between certain strains within *C. coli* and within *C. jejuni* and that these two species are indistinguishable using either gene^46^. The study found that the three regions were not able to produce fully robust phylogenetic trees for the *Campylobacter* genus, with the best produced tree using data from all three regions. Based on these findings, we would recommend caution when assessing phylogenetic relatedness of species based on limited genomic information. Intra-species differentiation was therefore undertaken using a whole genome based approach. Our findings of extremely limited intra-species genetic similarity within the *Campylobacter* genus perhaps explain why simplistic clustering based on single or selected genes is less effective than in some other genera.

Based on its status as an emerging pathogen, which has been associated with several diseases, and due to its ability to invade host cells, damage intestinal epithelial barriers, induce proinflammatory cytokine production, and form biofilms, we undertook an extensive genomic evaluation of the virulence factor profile of *C. concisus*. Microbe–host interactions are multi-factorial and may be achieved through many different mechanisms. These include secretion of immunomodulatory substances, production of surface molecules that mediate adhesion, and interaction with epithelial cells, or production of enzymes that modify the host extracellular matrix or cell surface receptors. We looked particularly for the presence of previously described virulence factors, including Zot and Exotoxin 9. Zot was first detected in *Vibrio cholerae* and is known to increase intestinal permeability through tight junction disruption^47^. More recently it has been shown to be prevalent in *C. concisus* isolates obtained from IBD patients^13,42^. Exotoxin 9 was originally described as a virulence factor for *C. concisus* isolates, as it appeared to differentiate chronic versus acute intestinal disease isolates. Exotoxin 9 was discovered within a 30-kb plasmid, which also contained a number of other virulence determinants. The original report indicated that the presence of the plasmid was only detected in highly invasive chronic intestinal disease strains, thus providing evidence of pathogenic potential. Both factors were identified in our study, with the majority of strains possessing one or both. Seventy-two percent of strains were Zot positive, which is in contrast to previously published findings from the southern hemisphere only, where only 30% of strains showed positivity^42^. Geographical differences in genetic expression may explain this difference and is worthy of specific study. We also found that GSII strains had higher rates of positivity for Exotoxin 9, compared with GSI, although this did not correlate with disease presentation or site of isolation – oral or faecal. Forty-five percent of isolates contained both virulence factors.

We also assessed the presence/absence of bacterial secretion systems focussing specifically on T4SS and T6SS, as these have been specifically interrogated within the *Campylobacter* genus. T4SS are transmembrane large protein complexes which traverse the cell envelope of many bacterial species and allow the delivery of effector molecules from the bacterium to the host. T4SS are seen in other members of the *Campylobacter* genus^48^. T4SSs are often encoded on self-transmissible plasmids, together with genes that provide selective advantage for the cell such as antibiotic resistance, virulence traits, or other metabolic functions that enhance survival. They can also be found as part of transposons integrated in chromosomes. We demonstrated that only 22% of *C. concisus* strains contained the T4SS, with both oral and colonic strains containing the T4SS machinery. The next step will be to experimentally validate our findings to allow us to elucidate the functional implications of the T4SS within *C. concisus*. It will also be interesting to determine whether there is any synergy between T4SS and T6SS secretion systems, as T4SS positive strains were also T6SS positive. T6SS are only present in Gram-negative bacterial genomes and are reminiscent of phage injection machinery^49^. The pathogenic potential of T6SS is well recognised in several human pathogens, including *V. cholera, Pseudomonas aeruginosa*, and *Francisella tularensis*^50–52^. T6SS have also been implicated in bacterial growth, motility, and survival under different stress conditions^49^. Although T6SS were first identified in *C. concisus* in 2011^12^, the present study allowed us for the first time to assess its presence/absence within a large number of genomes. Eighty-two percent of strains demonstrated the presence of T6SS, suggesting it may potentially confer some benefit in terms of host colonisation and virulence potential. The role and function of T6SS remains to be elucidated although studies suggest that T6SS carrying strains may selectively target proteobacterial commensals in order to facilitate competitive advantage over other potential colonisers^53^. Further studies looking to delineate the pathogenic potential of *C. concisus* strains including large-scale proteomic comparisons or mutation studies focussing on specific gene deletions including T4SS/T6SS or other virulence factors is now needed to gain mechanistic insight to the genome information that is now available. Other candidates include the other genes which were highlighted in the CARD and VFDB analysis. However, the lack of alignment highlighted that *C. concisus* was only distantly related to well-characterised pathogens. Bearing this in mind, transcriptomic/proteomic studies aimed at defining virulence markers would be beneficial to delve further into defining *C. concisus* virulence.

With the advent of improving bioinformatic tools, this is first study to analyse and report the genome and plasmid content of *C. concisus* isolates thus providing new insights into potential horizontally transmissible genetic elements that these strains may contain. As well as harbouring secretion system machinery within their genomes, many isolates had secretion systems IV and VI genes identified within their plasmids. The isolates with secretion system genes in their plasmids also had these genes in their genomes. There are a variety of reasons for this observation. Firstly, plasmids can be vertically transmitted from individual to individual, which creates a pathogenic community due to opportunism. In support of this, some *C. concisus* strains were shown to have genes present on their plasmids which enhanced virulence potential. It is also possible that pathogenic organisms may transfer virulence genes, through horizontal gene transfer (HGT), to *C. concisus*. In a study looking at *Campylobacter* infections, 10% of *C. concisus* infections were found to be co-infections with other enteropathogens such as *Clostridium difficile* and *Salmonella enterica*^2^. It is worth noting that *Salmonella typhimurium* can promote conjugative transfer of fitness-, virulence-, and antibiotic-resistance determinants to commensal *E. coli*^54^. Although *Salmonella* and *Escherichia*are closely related genera, this opens up the possibility that this could be a Proteobacterial specific trait and further work should be undertaken to fully understand how susceptible *C. concisus* strains are to HGT. Interspecies transfer of plasmids with antibiotic resistance genes has been demonstrated in children in rural India^55^. Commensal *E. coli* strains were shown to act as a reservoir of resistance and virulence genes that could be transferred between the same or different species. Plasmids are a burden on bacteria and so it would be advantageous for a community of commensal *C. concisus* to have only a few isolates present that contained plasmids, important for virulence that could then be transferred to other isolates during infection when the organisms have a high supply of nutrients.

The majority of isolates studied herein were from oral and faecal sources, and only a few strains were cultured from mucosal biopsies, which is a clear limitation. It is at present unknown if subgroups of *C. concisus* are selectively distributed between gut mucosa versus oral and faecal microbiota. Furthermore, isolates from healthy individuals were not included in our sequencing study. Additional comparative studies of strains from healthy and diseases individuals will be important to assess the genomics of disease pathogenesis. *C. concisus* strains may act normally as commensals, with previous studies showing *C. concisus* is a commensal within healthy oral site^56^, but can become an opportunistic pathogen, i.e., they are pathobionts (resident bacteria with pathogenic potential)^57^. Other commensal bacteria that can transition from commensal to pathogen include *Propionibacterium acnes*^58^ and *Candida albicans*^59^. It is therefore possible that dysbiosis of the gut, such as in response to infection and inflammation, may promote an increase in the rate of HGT thus enhancing the pathogenic potential of this organism^54^.

## Conclusion

This study reports the draught genome sequences of 52 new clinical *C. concisus* isolates and presents a comparative genomic analysis of currently available *C. concisus* genomes. The study highlights that the pathogenic potential of *C. concisus* is strain-specific and that virulence factor profiles do not stratify based on disease presentation or body site and that individuals can harbour multiple *C. concisus* strains concurrently. It is now clear that *C. concisus* can be present in virtually every part of the human GI tract and, under certain conditions, it can cause disease symptoms and also promote prolonged intestinal inflammation. There is therefore a need to further clarify the pathogenic potential of this relatively uncharacterised bacterial species.

## Electronic supplementary material

Supplementary material text

Supplementary Figure 1

Supplementary Figure 2

Supplementary Figure 3

Supplementary Figure 4

Supplementary Figure 5
