# Supplementary material for: Comparative genomics of Campylobacter concisus: Analysis of clinical strains reveals genome diversity and pathogenic potential
Source: Emerg Microbes Infect. 2018 Jun 26;7:116. doi: 10.1038/s41426-018-0118-x (PMC6018663; doi:10.1038/s41426-018-0118-x)
Supplement: Supplementary file 1 — Supplementary material text [file 41426_2018_118_MOESM1_ESM.docx]

**SUPPLEMENTAL MATERIALS FOR**

**Matthew R. Gemmell, Susan Berry, Indrani Mukhopadhya, Richard Hansen, Hans L. Nielsen, Mona Bajaj-Elliott, Henrik Nielsen, Georgina L. Hold, Comparative genomics of *Campylobacter concisus***

**This file contains:**

**Supplementary Material and Supplementary Tables**

**Supplementary Table 1: Genome statistics.** Quast results were calculated on contigs >= 500bp except where stated. The Illumina and PacBio coverage were calculated by aligning the Illumina MiSeq A5 error corrected reads and the PacBio-RSII filtered subreads respectively to the final assembly. The coverage for the Pacbio reads for samples B124-Slimy-small and B38-Tiny-mucoid were 174.60 and 695.57 respectively.

**Supplementary Table 2: Genome annotation**

**Supplementary Table 3: BUSCO results.** Assessment of the genome completeness with the use of Benchmarking Universal Single-Copy Orthologs (BUSCOs). BUSCOs were searched for in the amino acid fasta files created by Prokka. Proteobacteria BUSCO database was used for searching which contains 221 BUSCOs.

**Supplementary Table 4: REAPR genome assembly evaluation.** Assessment of errors within genome assemblies. FCD errors refers to Fragment Coverage Distribution errors based on the difference between the theoretical and observed FCD. FCD errors and low fragment coverage refer to regions that do not contain a gap.

**Supplementary Table 5: Comprehensive Antibiotic Resistance Database (CARD) and Virulence Factor Database (VFDB) presence in assembled genomes**

**Supplementary Table 6: Summary statistics for the pangenome of *Campylobacter concisus*. * GS = genomospecies**

**Supplementary Table 7: Summary statistics for the pangenome of *Campylobacter* species and *Campylobacter concisus*.** This was produced with roary using all the *C. concisus* genome assemblies used in this study and one reference assembly for every other *Campylobacter* species that was available. With the output files subsets of the pangenome were analysed.

**Supplementary Table 8: Plasmid assembly statistics.** Unless stated information is only for contigs/plasmids with a length greater than 500bp.

**Supplementary Table 9: Plasmid annotation statistics.**

**Supplementary Table 10: Isolates that share plasmid KEGG Orthology (ko0001) KEGG BRITE hierarchies within the different levels.**

**Supplementary Table 11: Summary statistics for the pangenome of *C. concisus* strains from oral faecal paired participant samples.**

**The following Supplementary Figures are supplied as separate files**

# Supplementary Figure 1: Overview of genome assemblies of *Campylobacter concisus* strains. Assessment of genome assemblies. Largest contig/Genome assembly was calculated by dividing the length of the largest contig by the total length of the genome assembly. N50/Genome assembly was calculated by dividing the length represented by the N50 by the total length of the genome assembly. Completeness represents the % complete estimation calculated by BUSCO analysis. Error free represents the % of the genome assembly that is error free estimation calculated by REAPR analysis.

# Supplementary Figure 2:  Phylogenetic tree, based on 23S rRNA of *C. concisus* strains used in this study, incorporating all published genomes. Exo9 and ZOT genome and plasmid presence/absence denoted on the 23S rRNA

Columns: Full stars represent Fecal samples, empty stars represent Oral samples. Squares represent presences in genome assemblies, Circles presence in plasmid assemblies. Red represents Exo9 whilst blue represents ZOT presence. Full shapes indicate presence, empty shapes indicate absence. For plasmids there are some samples with no shape, this indicates that plasmidspades was unable to assemble any plasmids for the sample.

**Supplementary Figure 3: Number of genes that are within different KEGG BRITE hierarchies in the pangenomes of different groups for the *C. concisus* samples.** Plot are separated by the KEGG BRITE hierarchy groups (A) Cellular Processes, (B) Environmental Information Processing, (C) Genetic Information Processing, (D) Human Diseases, (E) Metabolism and (F) Organismal Systems.

**Supplementary Figure 4: Number of genes that are within different KEGG BRITE hierarchies in the pangenomes of different groups for *Campylobacter* species and *C. concisus* samples.** Plot are separated by the KEGG BRITE hierarchy groups (A) Cellular Processes, (B) Environmental Information Processing, (C) Genetic Information Processing, (D) Human Diseases, (E) Metabolism and (F) Organismal Systems.

**Supplementary Figure 5: Pangenome summary of paired faecal/oral *C. concisus* isolates.** The figure displays how many genes were found to be shared across a certain percentage of samples in different groupings of isolates. The figure depicts a bin size of 20% on the x-axis.

**Supplementary Table 1**

| **Sample name** | **# contigs**  **(>= 0 bp)** | **Total length**  **(>= 0 bp)** | **# contigs** | **Largest contig (bp)** | **Total length (bp)** | **GC (%)** | **N50** | **N75** | **L50** | **L75** | **Genome coverage** |
| --- | --- | --- | --- | --- | --- | --- | --- | --- | --- | --- | --- |
| 2009-118452 | 50 | 2190058 | 50 | 269801 | 2190058 | 39.39 | 134300 | 113053 | 6 | 10 | 78.93 |
| 2009-119100 | 30 | 2170602 | 30 | 460743 | 2170602 | 39.41 | 412385 | 140455 | 3 | 5 | 87.33 |
| 2009-129008 | 45 | 2133657 | 45 | 690979 | 2133657 | 39.74 | 659079 | 200684 | 2 | 4 | 97.35 |
| 2009-130586 | 41 | 2074973 | 41 | 764112 | 2074973 | 39.48 | 499347 | 416462 | 2 | 3 | 109.10 |
| 2009-158448 | 24 | 1940298 | 24 | 363655 | 1940298 | 37.71 | 278217 | 137398 | 3 | 6 | 98.27 |
| 2009-173039 | 17 | 1903393 | 17 | 404697 | 1903393 | 37.73 | 315915 | 185157 | 3 | 5 | 113.55 |
| 2009-42653 | 13 | 2012410 | 13 | 667921 | 2012410 | 37.22 | 431996 | 197411 | 2 | 4 | 67.03 |
| 2009-75710 | 23 | 2093275 | 23 | 588562 | 2093275 | 39.51 | 538645 | 272757 | 2 | 4 | 73.37 |
| 2009-75775 | 52 | 2145119 | 52 | 461349 | 2145119 | 39.26 | 311624 | 147677 | 3 | 6 | 65.78 |
| 2009-86120 | 41 | 2055937 | 41 | 389167 | 2055937 | 39.6 | 175591 | 117488 | 4 | 7 | 91.51 |
| 2009-91522 | 12 | 2057910 | 12 | 1042741 | 2057910 | 39.5 | 1042741 | 334060 | 1 | 3 | 33.63 |
| 2010-112100-O | 12 | 2030392 | 12 | 967116 | 2030392 | 39.23 | 717940 | 717940 | 2 | 2 | 57.96 |
| 2010-112100-F | 20 | 1898749 | 20 | 847537 | 1898749 | 37.42 | 667365 | 667365 | 2 | 2 | 74.20 |
| 2010-112708 | 47 | 2133743 | 47 | 902212 | 2133743 | 39.62 | 355096 | 99127 | 2 | 5 | 90.77 |
| 2010-112758 | 29 | 2151215 | 29 | 1123368 | 2151215 | 39.38 | 1123368 | 510607 | 1 | 2 | 60.60 |
| 2010-112825 | 15 | 1913489 | 15 | 370081 | 1913489 | 39.67 | 301650 | 191352 | 3 | 5 | 77.04 |
| 2010-113332-F | 22 | 1982161 | 22 | 1050073 | 1982161 | 37.65 | 1050073 | 174136 | 1 | 4 | 106.06 |
| 2010-113332-O | 7 | 2069805 | 7 | 1173022 | 2069805 | 39.4 | 1173022 | 607976 | 1 | 2 | 59.36 |
| 2010-113862-O | 18 | 1951669 | 18 | 458778 | 1951669 | 39.66 | 414510 | 256570 | 3 | 4 | 85.96 |
| 2010-113862-F | 40 | 2005747 | 40 | 297258 | 2005747 | 39.81 | 171133 | 136645 | 5 | 8 | 79.57 |
| 2010-115605-F | 49 | 2145294 | 49 | 989329 | 2145294 | 39.36 | 331365 | 295605 | 2 | 3 | 102.20 |
| 2010-115605-O | 177 | 4423775 | 177 | 334763 | 4423775 | 39.11 | 81456 | 54010 | 16 | 32 | 39.77 |
| 2010-131105 | 7 | 2051300 | 7 | 1790271 | 2051300 | 39.29 | 1790271 | 1790271 | 1 | 1 | 67.75 |
| 2010-16206 | 14 | 1997691 | 14 | 505393 | 1997691 | 39.55 | 285855 | 196753 | 3 | 5 | 92.66 |
| 2010-164712 | 37 | 2057468 | 37 | 430218 | 2057468 | 37.28 | 209782 | 176354 | 4 | 6 | 112.34 |
| 2010-1718 | 33 | 1971591 | 33 | 360928 | 1971591 | 37.45 | 204383 | 187358 | 4 | 6 | 103.06 |
| 2012-191940 | 44 | 2205374 | 44 | 277524 | 2205374 | 39.36 | 213464 | 142285 | 5 | 8 | 84.18 |
| 2010-25654-F | 14 | 1885136 | 14 | 716714 | 1885136 | 37.59 | 644971 | 164910 | 2 | 3 | 93.35 |
| 2010-25654-O | 23 | 1898092 | 23 | 448575 | 1898092 | 37.64 | 222669 | 148815 | 3 | 6 | 98.03 |
| 2010-30795 | 41 | 2138161 | 41 | 805453 | 2138161 | 39.56 | 465091 | 390497 | 2 | 3 | 74.34 |
| 2010-30800 | 49 | 2123453 | 49 | 699973 | 2123453 | 39.47 | 279271 | 190019 | 3 | 5 | 100.62 |
| 2010-31374 | 19 | 1953836 | 19 | 527245 | 1953836 | 39.67 | 270122 | 246556 | 3 | 4 | 96.64 |
| 2010-33561 | 16 | 2128539 | 16 | 1176413 | 2128539 | 39.19 | 1176413 | 230063 | 1 | 3 | 62.76 |
| 2010-34330 | 11 | 2154818 | 11 | 812842 | 2154818 | 39.37 | 704168 | 519905 | 2 | 3 | 69.52 |
| 2010-347972 | 73 | 2059946 | 73 | 318634 | 2059946 | 37.77 | 170299 | 84718 | 5 | 9 | 35.92 |
| 2010-36743 | 13 | 2104638 | 13 | 1361737 | 2104638 | 39.38 | 1361737 | 432100 | 1 | 2 | 71.05 |
| 2010-378007-F | 23 | 1848443 | 23 | 499289 | 1848443 | 37.42 | 276206 | 199355 | 3 | 5 | 96.88 |
| 2010-378007-O | 12 | 1858622 | 12 | 808538 | 1858622 | 37.37 | 259737 | 200741 | 2 | 4 | 53.59 |
| 2010-43100 | 43 | 2136656 | 43 | 953814 | 2136656 | 39.3 | 539813 | 245148 | 2 | 3 | 71.43 |
| 2010-6073 | 26 | 1953194 | 26 | 589543 | 1953194 | 37.53 | 190430 | 165687 | 3 | 6 | 103.80 |
| 2010-8194 | 33 | 1920703 | 33 | 343633 | 1920703 | 37.54 | 195268 | 124799 | 4 | 8 | 92.57 |
| 2010-88823 | 41 | 2105787 | 41 | 544762 | 2105787 | 39.49 | 206342 | 104647 | 4 | 7 | 86.58 |
| 2012-164712 | 16 | 2032479 | 16 | 443979 | 2032479 | 37.22 | 314582 | 264763 | 3 | 5 | 32.02 |
| 2012-37302 | 40 | 1948687 | 40 | 303326 | 1948687 | 37.45 | 155627 | 121037 | 5 | 8 | 94.88 |
| 2013-101463 | 32 | 1956374 | 32 | 409663 | 1956374 | 39.58 | 275014 | 171104 | 3 | 6 | 70.91 |
| 2013-39845 | 45 | 2165919 | 45 | 836769 | 2165919 | 39.37 | 739931 | 339212 | 2 | 3 | 89.91 |
| 2013-42088 | 19 | 2051445 | 19 | 458228 | 2051445 | 39.3 | 324487 | 215828 | 3 | 5 | 85.45 |
| 2013-87946 | 31 | 1896326 | 31 | 389325 | 1896326 | 37.79 | 141731 | 110081 | 5 | 8 | 91.99 |
| B124_Small-clear | 18 | 2046128 | 18 | 429067 | 2046128 | 39.34 | 345462 | 250287 | 3 | 5 | 84.70 |
| B124_Small-grey | 5 | 2048069 | 5 | 1422724 | 2048069 | 39.45 | 1422724 | 556700 | 1 | 2 | 74.16 |
| B38_Tiny-mucoid | 143 | 2288052 | 143 | 680624 | 2288052 | 39.5 | 475808 | 262150 | 2 | 4 | 52.52 |
| B124_Slimy-large | 32 | 2133351 | 32 | 554781 | 2133351 | 39.42 | 442012 | 297935 | 3 | 4 | 63.31 |
| B124_Slimy-small | 11 | 2090450 | 11 | 886017 | 2090450 | 39.22 | 827044 | 827044 | 2 | 2 | 74.89 |

**Supplementary Table 2**

| **Sample name** | **# bases** | **CDS** | **contigs** | **# genes** | **# repeat regions** | **# tmRNA** | **# tRNA** |
| --- | --- | --- | --- | --- | --- | --- | --- |
| 2009-118452 | 2190058 | 1967 | 50 | 2011 | 2 | 1 | 41 |
| 2009-119100 | 2170602 | 2013 | 30 | 2063 | 3 | 1 | 43 |
| 2009-129008 | 2133657 | 1950 | 45 | 1991 | 1 | 1 | 38 |
| 2009-130586 | 2074973 | 1959 | 41 | 2000 | 0 | 1 | 38 |
| 2009-158448 | 1940298 | 1786 | 24 | 1832 | 0 | 1 | 43 |
| 2009-173039 | 1903393 | 1882 | 17 | 1926 | 0 | 1 | 41 |
| 2009-42653 | 2012410 | 1978 | 13 | 2024 | 2 | 1 | 43 |
| 2009-75710 | 2093275 | 1897 | 23 | 1941 | 1 | 1 | 41 |
| 2009-75775 | 2145119 | 2020 | 52 | 2065 | 3 | 1 | 42 |
| 2009-86120 | 2055937 | 1939 | 41 | 1981 | 1 | 1 | 39 |
| 2009-91522 | 2057910 | 1990 | 12 | 2034 | 6 | 1 | 41 |
| 2010-112100-O | 2030392 | 1946 | 12 | 1996 | 1 | 1 | 45 |
| 2010-112100-F | 1898749 | 1826 | 20 | 1870 | 0 | 1 | 41 |
| 2010-112708 | 2133743 | 1953 | 47 | 2002 | 1 | 1 | 46 |
| 2010-112758 | 2151215 | 2018 | 29 | 2065 | 2 | 1 | 42 |
| 2010-112825 | 1913489 | 1869 | 15 | 1923 | 0 | 1 | 47 |
| 2010-113332-F | 1982161 | 1876 | 22 | 1924 | 2 | 1 | 45 |
| 2010-113332-O | 2069805 | 1976 | 7 | 2020 | 3 | 1 | 41 |
| 2010-113862-O | 1951669 | 1947 | 18 | 1991 | 0 | 1 | 41 |
| 2010-113862-F | 2005747 | 1911 | 40 | 1952 | 0 | 1 | 38 |
| 2010-115605-F | 2145294 | 1961 | 49 | 2007 | 1 | 1 | 41 |
| 2010-131105 | 2051300 | 1972 | 7 | 2018 | 1 | 1 | 43 |
| 2010-16206 | 1997691 | 1923 | 14 | 1968 | 0 | 1 | 42 |
| 2010-164712 | 2057468 | 2006 | 37 | 2055 | 3 | 1 | 46 |
| 2010-1718 | 1971591 | 1788 | 33 | 1832 | 0 | 1 | 41 |
| 2010-25654-F | 1885136 | 1839 | 14 | 1886 | 0 | 1 | 44 |
| 2010-25654-O | 1898092 | 1828 | 23 | 1876 | 0 | 1 | 45 |
| 2010-30795 | 2138161 | 1946 | 41 | 1993 | 2 | 1 | 44 |
| 2010-30800 | 2123453 | 1944 | 49 | 1992 | 2 | 1 | 45 |
| 2010-31374 | 1953836 | 1853 | 19 | 1899 | 1 | 1 | 43 |
| 2010-33561 | 2128539 | 2044 | 16 | 2097 | 3 | 1 | 49 |
| 2010-34330 | 2154818 | 2010 | 11 | 2053 | 3 | 1 | 40 |
| 2010-347972 | 2059946 | 1941 | 73 | 1986 | 1 | 1 | 40 |
| 2010-36743 | 2104638 | 1997 | 13 | 2041 | 3 | 1 | 41 |
| 2010-378007-F | 1848443 | 1778 | 23 | 1819 | 2 | 1 | 38 |
| 2010-378007-O | 1858622 | 1793 | 12 | 1837 | 2 | 1 | 41 |
| 2010-43100 | 2136656 | 1990 | 43 | 2032 | 2 | 1 | 39 |
| 2010-6073 | 1953194 | 1818 | 26 | 1864 | 0 | 1 | 43 |
| 2010-8194 | 1920703 | 1782 | 33 | 1832 | 0 | 1 | 47 |
| 2010-88823 | 2105787 | 1904 | 41 | 1947 | 1 | 1 | 40 |
| 2012-164712 | 2032479 | 2014 | 16 | 2059 | 2 | 1 | 42 |
| 2012-191940 | 2205374 | 1984 | 44 | 2034 | 6 | 2 | 44 |
| 2012-37302 | 1948687 | 1783 | 40 | 1827 | 0 | 0 | 42 |
| 2013-101463 | 1956374 | 1921 | 32 | 1965 | 0 | 1 | 41 |
| 2013-39845 | 2165919 | 2009 | 45 | 2049 | 2 | 1 | 37 |
| 2013-42088 | 2051445 | 1957 | 19 | 1995 | 1 | 1 | 35 |
| 2013-87946 | 1896326 | 1768 | 31 | 1812 | 0 | 1 | 41 |
| B124-slimy-large | 2288052 | 2075 | 143 | 2119 | 2 | 1 | 39 |
| B124-slimy-small | 2090450 | 2019 | 11 | 2064 | 1 | 1 | 42 |
| B124-small-clear | 2133351 | 1988 | 32 | 2040 | 1 | 1 | 45 |
| B124-Small-grey | 2046128 | 1979 | 18 | 2021 | 1 | 1 | 39 |
| B38_Tiny-mucoid | 2048069 | 1967 | 5 | 2009 | 1 | 1 | 39 |

**Supplementary Table 3**

| **Sample name** | **Complete BUSCOs** | **Complete and single-copy BUSCOs** | **Complete and duplicated BUSCOs** | **Fragmented BUSCOs** | **Missing BUSCOs** |
| --- | --- | --- | --- | --- | --- |
| 2009-118452 | 201 | 200 | 1 | 6 | 14 |
| 2009-119100 | 203 | 203 | 0 | 4 | 14 |
| 2009-129008 | 205 | 205 | 0 | 3 | 13 |
| 2009-130586 | 203 | 203 | 0 | 4 | 14 |
| 2009-158448 | 201 | 200 | 1 | 5 | 15 |
| 2009-173039 | 202 | 202 | 0 | 3 | 16 |
| 2009-42653 | 204 | 204 | 0 | 4 | 13 |
| 2009-75710 | 205 | 205 | 0 | 3 | 13 |
| 2009-75775 | 202 | 202 | 0 | 4 | 15 |
| 2009-86120 | 203 | 201 | 2 | 4 | 14 |
| 2009-91522 | 205 | 205 | 0 | 3 | 13 |
| 2010-112100-O | 205 | 205 | 0 | 3 | 13 |
| 2010-112100-F | 205 | 205 | 0 | 2 | 14 |
| 2010-112708 | 201 | 195 | 6 | 5 | 15 |
| 2010-112758 | 202 | 201 | 1 | 4 | 15 |
| 2010-112825 | 204 | 204 | 0 | 3 | 14 |
| 2010-113332-F | 202 | 200 | 2 | 5 | 14 |
| 2010-113332-O | 204 | 204 | 0 | 4 | 13 |
| 2010-113862-O | 205 | 205 | 0 | 3 | 13 |
| 2010-113862-F | 204 | 202 | 2 | 4 | 13 |
| 2010-115605-F | 203 | 201 | 2 | 3 | 15 |
| 2010-131105 | 203 | 203 | 0 | 3 | 15 |
| 2010-16206 | 203 | 203 | 0 | 5 | 13 |
| 2010-164712 | 205 | 205 | 0 | 2 | 14 |
| 2010-1718 | 201 | 201 | 0 | 4 | 16 |
| 2010-25654-F | 205 | 205 | 0 | 4 | 12 |
| 2010-25654-O | 203 | 203 | 0 | 5 | 13 |
| 2010-30795 | 204 | 202 | 2 | 4 | 13 |
| 2010-30800 | 205 | 205 | 0 | 3 | 13 |
| 2010-31374 | 205 | 205 | 0 | 3 | 13 |
| 2010-33561 | 205 | 205 | 0 | 3 | 13 |
| 2010-34330 | 201 | 201 | 0 | 5 | 15 |
| 2010-347972 | 201 | 201 | 0 | 6 | 14 |
| 2010-36743 | 203 | 203 | 0 | 4 | 14 |
| 2010-378007-F | 204 | 204 | 0 | 4 | 13 |
| 2010-378007-O | 204 | 204 | 0 | 4 | 13 |
| 2010-43100 | 204 | 204 | 0 | 4 | 13 |
| 2010-6073 | 202 | 202 | 0 | 4 | 15 |
| 2010-8194 | 201 | 201 | 0 | 5 | 15 |
| 2010-88823 | 204 | 204 | 0 | 4 | 13 |
| 2012-164712 | 205 | 205 | 0 | 2 | 14 |
| 2012-191940 | 202 | 199 | 3 | 5 | 14 |
| 2012-37302 | 202 | 202 | 0 | 5 | 14 |
| 2013-101463 | 202 | 202 | 0 | 5 | 14 |
| 2013-39845 | 202 | 202 | 0 | 5 | 14 |
| 2013-42088 | 205 | 205 | 0 | 3 | 13 |
| 2013-87946 | 202 | 202 | 0 | 5 | 14 |
| B124-slimy-large | 201 | 201 | 0 | 4 | 16 |
| B124-slimy-small | 203 | 203 | 0 | 4 | 14 |
| B124-small-clear | 205 | 205 | 0 | 3 | 13 |
| B124-Small-grey | 205 | 205 | 0 | 3 | 13 |
| B38_Tiny-mucoid | 204 | 204 | 0 | 3 | 14 |

**Supplementary Table 4: REAPR genome assembly evaluation.** Assessment of errors within genome assemblies. FCD errors refers to Fragment Coverage Distribution errors based on the difference between the theoretical and observed FCD. FCD errors and low fragment coverage refer to regions that do not contain a gap. Due to contamination within the reads, reapr estimation for 2010-347972 was not carried out.

| **Sample name** | **Bases** | **Error free Bases** | **#Gaps** | **# Bases in gaps (i.e. Ns)** | **FCD errors** | **FCD errors over a gap** | **Low fragment coverage** | **Low fragment coverage over a gap** |
| --- | --- | --- | --- | --- | --- | --- | --- | --- |
| 2009-118452 | 2190058 | 1776988 | 96 | 137708 | 9 | 0 | 2814 | 22 |
| 2009-119100 | 2170602 | 1805393 | 101 | 103640 | 3 | 0 | 3862 | 27 |
| 2009-129008 | 2133657 | 1720981 | 154 | 131881 | 2 | 0 | 3749 | 45 |
| 2009-130586 | 2074973 | 1731333 | 109 | 76014 | 4 | 0 | 2878 | 29 |
| 2009-158448 | 1940298 | 1505554 | 103 | 141097 | 0 | 0 | 4509 | 26 |
| 2009-173039 | 1903393 | 1690636 | 36 | 41971 | 11 | 0 | 2573 | 11 |
| 2009-42653 | 2012410 | 1827586 | 31 | 48328 | 3 | 0 | 2787 | 7 |
| 2009-75710 | 2093275 | 1771733 | 76 | 134745 | 6 | 0 | 2436 | 15 |
| 2009-75775 | 2145119 | 1915871 | 56 | 57367 | 8 | 0 | 1682 | 11 |
| 2009-86120 | 2055937 | 1764660 | 79 | 92389 | 2 | 1 | 2438 | 18 |
| 2009-91522 | 2057910 | 1649436 | 63 | 35031 | 2 | 2 | 2629 | 14 |
| 2010-112100-F | 1898749 | 1625615 | 29 | 60128 | 3 | 0 | 4744 | 6 |
| 2010-112100-O | 2030392 | 1895845 | 23 | 29076 | 10 | 0 | 3179 | 5 |
| 2010-112708 | 2133743 | 1598692 | 138 | 176313 | 1 | 0 | 3601 | 49 |
| 2010-112758 | 2151215 | 1922085 | 50 | 59085 | 6 | 1 | 2936 | 8 |
| 2010-112825 | 1913489 | 1679182 | 37 | 23024 | 23 | 1 | 1860 | 9 |
| 2010-113332-F | 1982161 | 1518999 | 79 | 101611 | 0 | 0 | 4289 | 19 |
| 2010-113332-O | 2069805 | 1904926 | 32 | 38696 | 9 | 0 | 2443 | 7 |
| 2010-113862 | 2005747 | 1673455 | 100 | 141314 | 1 | 0 | 2202 | 19 |
| 2010-113862-O | 1951669 | 1692758 | 40 | 43852 | 20 | 1 | 1702 | 10 |
| 2010-115605-F | 2145294 | 1781617 | 98 | 109053 | 3 | 0 | 3374 | 19 |
| 2010-131105 | 2051300 | 1942020 | 27 | 23292 | 15 | 0 | 1766 | 3 |
| 2010-16206 | 1997691 | 1888711 | 40 | 26814 | 5 | 0 | 1486 | 7 |
| 2010-164712 | 2057468 | 1727308 | 54 | 79164 | 0 | 0 | 4532 | 11 |
| 2010-1718 | 1971591 | 1596425 | 74 | 146682 | 0 | 0 | 3373 | 22 |
| 2010-25654-F | 1885136 | 1743431 | 25 | 44900 | 11 | 0 | 1490 | 3 |
| 2010-25654-O | 1898092 | 1644488 | 56 | 61600 | 2 | 1 | 3085 | 12 |
| 2010-30795 | 2138161 | 1798866 | 105 | 120647 | 7 | 1 | 2360 | 25 |
| 2010-30800 | 2123453 | 1745753 | 94 | 143811 | 1 | 3 | 2707 | 21 |
| 2010-31374 | 1953836 | 1730613 | 50 | 48121 | 3 | 0 | 1990 | 14 |
| 2010-33561 | 2128539 | 1940651 | 39 | 47045 | 12 | 0 | 2242 | 5 |
| 2010-34330 | 2154818 | 1963717 | 51 | 71550 | 2 | 0 | 3108 | 7 |
| 2010-347972 | NA | NA | NA | NA | NA | NA | NA | NA |
| 2010-36743 | 2104638 | 1952452 | 41 | 46611 | 7 | 0 | 3099 | 8 |
| 2010-378007-F | 1848443 | 1657884 | 48 | 40393 | 0 | 0 | 2963 | 10 |
| 2010-378007-O | 1858622 | 1676198 | 17 | 44340 | 4 | 0 | 2202 | 1 |
| 2010-43100 | 2136656 | 1887782 | 70 | 80483 | 9 | 2 | 1903 | 19 |
| 2010-6073 | 1953194 | 1565787 | 69 | 111490 | 1 | 0 | 3039 | 13 |
| 2010-8194 | 1920703 | 1612259 | 68 | 124525 | 3 | 0 | 3076 | 18 |
| 2010-88823 | 2105787 | 1752183 | 98 | 120747 | 4 | 0 | 2664 | 30 |
| 2012-164712 | 2032479 | 1811561 | 21 | 49451 | 3 | 0 | 2286 | 4 |
| 2012-191940 | 2205374 | 1714850 | 127 | 141223 | 2 | 0 | 3326 | 34 |
| 2012-37302 | 1948687 | 1610695 | 85 | 114365 | 4 | 1 | 2524 | 16 |
| 2013-101463 | 1956374 | 1741216 | 49 | 52878 | 12 | 2 | 1244 | 11 |
| 2013-39845 | 2165919 | 1825368 | 109 | 77987 | 11 | 2 | 2946 | 29 |
| 2013-42088 | 2051445 | 1796623 | 36 | 66621 | 5 | 0 | 4134 | 4 |
| 2013-87946 | 1896326 | 1588032 | 81 | 114017 | 2 | 0 | 2991 | 18 |
| B124_Slimy-small | 2090450 | 1840118 | 533 | 24274 | 3 | 0 | 3800 | 129 |
| B124_Small-clear | 2133351 | 1783632 | 93 | 97951 | 1 | 1 | 3943 | 22 |
| B124_Small-grey | 2046128 | 1883435 | 41 | 33463 | 22 | 0 | 2388 | 12 |
| B38_Tiny-mucoid | 2048069 | 1867140 | 41 | 23575 | 4 | 0 | 3874 | 14 |
| B124_Slimy-large | 2288052 | 1731971 | 142 | 120154 | 3 | 1 | 4433 | 52 |

**Supplementary Table 5**

|  | Genome | | |
| --- | --- | --- | --- |
|  | CARDs | VFDBs | |
|  |  | core set | all set |
| 2009-118452 | 7 | 4 | 20 |
| 2009-119100 | 7 | 2 | 22 |
| 2009-129008 | 7 | 2 | 12 |
| 2009-130586 | 7 | 2 | 18 |
| 2009-158448 | 7 | 4 | 28 |
| 2009-173039 | 7 | 2 | 14 |
| 2009-42653 | 7 | 4 | 26 |
| 2009-75710 | 7 | 2 | 22 |
| 2009-75775 | 7 | 0 | 8 |
| 2009-86120 | 7 | 2 | 26 |
| 2009-91522 | 7 | 2 | 16 |
| 2010-112100-F | 7 | 4 | 12 |
| 2010-112100-O | 7 | 2 | 16 |
| 2010-112708 | 7 | 6 | 28 |
| 2010-112758 | 7 | 4 | 14 |
| 2010-112825 | 7 | 2 | 12 |
| 2010-113332-F | 7 | 2 | 18 |
| 2010-113332-O | 7 | 4 | 26 |
| 2010-113862-F | 7 | 4 | 20 |
| 2010-113862-O | 7 | 4 | 20 |
| 2010-115605-F | 7 | 0 | 20 |
| 2010-131105 | 7 | 4 | 20 |
| 2010-16206 | 7 | 4 | 24 |
| 2010-164712 | 7 | 4 | 26 |
| 2010-1718 | 7 | 0 | 2 |
| 2010-25654-F | 7 | 4 | 30 |
| 2010-25654-O | 7 | 4 | 30 |
| 2010-30795 | 7 | 2 | 14 |
| 2010-30800 | 7 | 2 | 8 |
| 2010-31374 | 7 | 4 | 24 |
| 2010-33561 | 7 | 2 | 22 |
| 2010-34330 | 7 | 4 | 16 |
| 2010-347972 | 7 | 1 | 10 |
| 2010-36743 | 7 | 4 | 16 |
| 2010-378007-F | 7 | 0 | 10 |
| 2010-378007-O | 7 | 0 | 10 |
| 2010-43100 | 7 | 6 | 26 |
| 2010-6073 | 7 | 6 | 30 |
| 2010-8194 | 7 | 4 | 26 |
| 2010-88823 | 7 | 4 | 16 |
| 2012-164712 | 7 | 4 | 26 |
| 2012-191940 | 7 | 6 | 34 |
| 2012-37302 | 7 | 2 | 40 |
| 2013-101463 | 7 | 4 | 24 |
| 2013-39845 | 7 | 4 | 18 |
| 2013-42088 | 7 | 4 | 16 |
| 2013-87946 | 7 | 4 | 24 |
| B124_Slimy-small | 7 | 2 | 32 |
| B124_Small-clear | 7 | 2 | 12 |
| B124_Small-grey | 7 | 2 | 14 |
| B38_Tiny-mucoid | 7 | 4 | 16 |
| B124_Slimy-large | 7 | 2 | 30 |
| 13826 | 7 | 2 | 14 |
| ATCC_33237 | 7 | 2 | 14 |
| ATCC_51561 | 14 | 8 | 52 |
| ATCC_51562 | 14 | 12 | 72 |
| UNSW1 | 0 | 8 | 40 |
| UNSW2 | 0 | 4 | 44 |
| UNSW3 | 0 | 8 | 44 |
| UNSWCD | 0 | 4 | 44 |
| UNSWCS | 0 | 2 | 16 |
| H14O-S1 | 7 | 1 | 11 |
| H17O-S1 | 7 | 2 | 17 |
| H1O1 | 7 | 1 | 11 |
| H21O-S1 | 8 | 1 | 10 |
| H21O-S2 | 8 | 1 | 5 |
| H21O-S3 | 7 | 1 | 15 |
| H21O-S5 | 7 | 1 | 5 |
| H22O-S1 | 7 | 2 | 10 |
| H23O-S1 | 8 | 1 | 11 |
| H9O-S2 | 8 | 0 | 18 |
| P13UCO-S3 | 7 | 1 | 12 |
| P15UCO-S2 | 7 | 1 | 4 |
| P20CDO-S1 | 7 | 2 | 10 |
| P20CDO-S2 | 7 | 3 | 6 |
| P20CDO-S3 | 7 | 2 | 10 |
| P20CDO-S4 | 7 | 0 | 3 |
| P21CDO-S1 | 7 | 3 | 15 |
| P21CDO-S2 | 7 | 3 | 13 |
| P21CDO-S4 | 7 | 3 | 18 |
| P24CDO-S2 | 7 | 2 | 8 |
| P24CDO-S3 | 7 | 2 | 8 |
| P24CDO-S4 | 7 | 2 | 8 |
| P2CDO3 | 7 | 2 | 12 |
| P2CDO4 | 7 | 2 | 12 |
| P2CDO-S6 | 7 | 2 | 12 |
| P3UCB1 | 7 | 1 | 14 |
| P3UCO1 | 7 | 1 | 14 |

**Supplementary table 6:**

|  | **All Genomes** | **This study** | **Currently Public available samples** | **GS* I** | **GS II** | **GS I only** | **GS II only** |
| --- | --- | --- | --- | --- | --- | --- | --- |
| **Core genes (99% <= strains <= 100%)** | 541 | 563 | 587 | 600 | 552 | 0 | 0 |
| **Soft core genes (95% <= strains < 99%)** | 97 | 75 | 38 | 54 | 90 | 0 | 0 |
| **Shell genes (15% <= strains < 95%)** | 2313 | 2434 | 2253 | 2434 | 2324 | 5 | 2 |
| **Cloud genes (0% <= strains < 15%)** | 11576 | 7970 | 6562 | 5119 | 9746 | 1810 | 6318 |
| **Total genes** | 14527 | 11042 | 9440 | 8207 | 12712 | 1815 | 6320 |

**Supplementary table 7**

|  | **All Campylobacter** | **Other *Campylobacter*** | ***C. concisus*** | **Genes present in other *Campylobacter* and absent in *C. concisus*** | **Genes present in *C. concisus* and absent in other *Campylobacter*** |
| --- | --- | --- | --- | --- | --- |
| **Core genes (99% <= strains <= 100%)** | 3 | 2 | 9 | 0 | 0 |
| **Soft core genes (95% <= strains < 99%)** | 5 | 0 | 615 | 0 | 9 |
| **Shell genes (15% <= strains < 95%)** | 2679 | 431 | 2320 | 217 | 1419 |
| **Cloud genes (0% <= strains < 15%)** | 42917 | 31360 | 13083 | 29363 | 11132 |
| **Total genes** | 45604 | 31793 | 16027 | 29580 | 12560 |

**Supplementary Table 8**

| **Sample name** | **# contigs (>= 0 bp)** | **Total length (>= 0 bp)** | **# contigs** | **Largest contig** | **Total length** | **GC (%)** | **N50** | **N75** | **L50** | **L75** |
| --- | --- | --- | --- | --- | --- | --- | --- | --- | --- | --- |
| 2009-118452 | 3 | 16823 | 1 | 16526 | 16526 | 33.45 | 16526 | 16526 | 1 | 1 |
| 2009-119100 | 10 | 33934 | 6 | 18118 | 32904 | 36.79 | 18118 | 7122 | 1 | 2 |
| 2009-129008 | 7 | 91543 | 7 | 24466 | 91543 | 36.14 | 13235 | 11719 | 3 | 5 |
| 2009-130586 | 5 | 45925 | 5 | 17254 | 45925 | 33.21 | 14880 | 10482 | 2 | 3 |
| 2009-158448 | 1 | 11944 | 1 | 11944 | 11944 | 32.4 | 11944 | 11944 | 1 | 1 |
| 2009-173039 | 10 | 98427 | 7 | 36433 | 97754 | 36.81 | 26373 | 14170 | 2 | 3 |
| 2009-42653 | 2 | 76072 | 2 | 41129 | 76072 | 35.35 | 41129 | 34943 | 1 | 2 |
| 2009-75775 | 5 | 103727 | 4 | 49148 | 103418 | 37.68 | 25282 | 24349 | 2 | 3 |
| 2009-86120 | 3 | 30909 | 3 | 16327 | 30909 | 34.02 | 16327 | 13292 | 1 | 2 |
| 2009-91522 | 128 | 100725 | 20 | 18964 | 78739 | 37.72 | 10923 | 4835 | 3 | 6 |
| 2010-112100-F | 1 | 35370 | 1 | 35370 | 35370 | 32.85 | 35370 | 35370 | 1 | 1 |
| 2010-112100-O | 52 | 96410 | 20 | 17663 | 88086 | 34.02 | 7582 | 4501 | 4 | 8 |
| 2010-112708 | 2 | 12861 | 2 | 11398 | 12861 | 32.41 | 11398 | 11398 | 1 | 1 |
| 2010-112758 | 6 | 99371 | 5 | 61402 | 99116 | 34.05 | 61402 | 21972 | 1 | 2 |
| 2010-112825 | 39 | 121971 | 23 | 16511 | 117704 | 38.5 | 10747 | 8933 | 5 | 8 |
| 2010-113332-F | 3 | 81434 | 3 | 66819 | 81434 | 38.64 | 66819 | 66819 | 1 | 1 |
| 2010-113332-O | 61 | 157295 | 27 | 30754 | 148406 | 37.25 | 8599 | 4234 | 4 | 9 |
| 2010-113862-F | 4 | 45139 | 4 | 18229 | 45139 | 36.21 | 15083 | 10311 | 2 | 3 |
| 2010-113862-O | 8 | 19088 | 6 | 7375 | 18664 | 36.73 | 4019 | 3042 | 2 | 3 |
| 2010-115605-F | 60 | 129124 | 21 | 16061 | 118898 | 39.17 | 11674 | 5253 | 5 | 8 |
| 2010-164712 | 1 | 10852 | 1 | 10852 | 10852 | 30.95 | 10852 | 10852 | 1 | 1 |
| 2010-1718 | 5 | 73907 | 5 | 32022 | 73907 | 33 | 15150 | 12123 | 2 | 3 |
| 2010-25654-F | 6 | 61396 | 3 | 31060 | 60650 | 33.78 | 31060 | 16144 | 1 | 2 |
| 2010-25654-O | 3 | 46668 | 3 | 22079 | 46668 | 33.58 | 13446 | 13446 | 2 | 2 |
| 2010-30795 | 3 | 31499 | 3 | 15910 | 31499 | 36.24 | 15910 | 11611 | 1 | 2 |
| 2010-30800 | 3 | 13734 | 1 | 13437 | 13437 | 33.82 | 13437 | 13437 | 1 | 1 |
| 2010-33561 | 38 | 123474 | 13 | 21474 | 117640 | 40.67 | 17052 | 11148 | 3 | 6 |
| 2010-34330 | 8 | 44086 | 6 | 25378 | 43454 | 37.08 | 25378 | 11331 | 1 | 2 |
| 2010-36743 | 5 | 39606 | 4 | 18684 | 39386 | 36.02 | 13024 | 13024 | 2 | 2 |
| 2010-378007-F | 4 | 49811 | 4 | 17829 | 49811 | 32.73 | 15937 | 12800 | 2 | 3 |
| 2010-378007-O | 77 | 389850 | 32 | 43513 | 378936 | 38.17 | 16319 | 11960 | 7 | 14 |
| 2010-43100 | 29 | 180604 | 19 | 28806 | 177978 | 34 | 12098 | 9479 | 5 | 9 |
| 2010-8194 | 2 | 23265 | 2 | 18566 | 23265 | 31.85 | 18566 | 18566 | 1 | 1 |
| 2010-88823 | 7 | 57782 | 7 | 19255 | 57782 | 33.43 | 16597 | 12605 | 2 | 3 |
| 2012-164712 | 1 | 34899 | 1 | 34899 | 34899 | 38.38 | 34899 | 34899 | 1 | 1 |
| 2012-191940 | 9 | 88273 | 9 | 42012 | 88273 | 33.77 | 12475 | 10784 | 2 | 4 |
| 2012-37302 | 2 | 27257 | 2 | 14118 | 27257 | 36.13 | 14118 | 13139 | 1 | 2 |
| 2013-101463 | 3 | 41717 | 3 | 23982 | 41717 | 36.05 | 23982 | 11539 | 1 | 2 |
| 2013-39845 | 11 | 50780 | 10 | 21449 | 50520 | 36.08 | 12939 | 5204 | 2 | 3 |
| 2013-42088 | 2 | 19757 | 1 | 19534 | 19534 | 33.71 | 19534 | 19534 | 1 | 1 |
| 2013-87946 | 2 | 25812 | 2 | 14148 | 25812 | 35.42 | 14148 | 11664 | 1 | 2 |
| B124_Slimy-small | 8 | 81333 | 6 | 19516 | 81036 | 34.27 | 13436 | 10563 | 3 | 5 |
| B124_Small-clear | 9 | 52718 | 6 | 18301 | 52073 | 36.39 | 13617 | 13436 | 2 | 3 |
| B124_Small-grey | 33 | 96610 | 18 | 13476 | 92856 | 34.4 | 11150 | 10299 | 4 | 6 |
| B38_Tiny-mucoid | 11 | 48049 | 9 | 14894 | 47276 | 35.94 | 11853 | 5211 | 2 | 4 |
| B124_Slimy-large | 23 | 107372 | 20 | 22362 | 106878 | 38.31 | 13436 | 6190 | 3 | 6 |
| H14O-S1 | 2 | 42505 | 2 | 32108 | 42505 | 32.63 | 32108 | 32108 | 1 | 1 |
| H17O-S1 | 2 | 71627 | 2 | 60918 | 71627 | 33.61 | 60918 | 60918 | 1 | 1 |
| H1O1 | 3 | 20198 | 2 | 10295 | 19726 | 32.56 | 10295 | 9431 | 1 | 2 |
| H21O-S1 | 4 | 59772 | 4 | 34863 | 59772 | 34.45 | 34863 | 11709 | 1 | 2 |
| H21O-S2 | 13 | 63765 | 5 | 33667 | 61871 | 39.48 | 33667 | 13062 | 1 | 2 |
| H21O-S3 | 2 | 56635 | 2 | 36824 | 56635 | 37.58 | 36824 | 19811 | 1 | 2 |
| H21O-S5 | 13 | 178260 | 9 | 55625 | 177556 | 35.7 | 39571 | 14364 | 2 | 4 |
| H22O-S1 | 13 | 102760 | 13 | 21617 | 102760 | 34.66 | 14202 | 12378 | 3 | 5 |
| H23O-S1 | 3 | 38998 | 3 | 19731 | 38998 | 34.74 | 19731 | 17742 | 1 | 2 |
| H9O-S2 | 1 | 3970 | 1 | 3970 | 3970 | 37.78 | 3970 | 3970 | 1 | 1 |
| P13UCO-S3 | 12 | 86961 | 9 | 43101 | 86132 | 36.27 | 43101 | 6066 | 1 | 3 |
| P15UCO-S2 | 4 | 47862 | 4 | 25100 | 47862 | 34.87 | 25100 | 19826 | 1 | 2 |
| P20CDO-S1 | 5 | 33544 | 4 | 15544 | 33156 | 35.02 | 11216 | 11216 | 2 | 2 |
| P20CDO-S2 | 5 | 154359 | 4 | 65837 | 154104 | 33.73 | 50676 | 50676 | 2 | 2 |
| P20CDO-S3 | 13 | 166069 | 6 | 116665 | 164717 | 32.86 | 116665 | 18106 | 1 | 2 |
| P20CDO-S4 | 1 | 29033 | 1 | 29033 | 29033 | 30.08 | 29033 | 29033 | 1 | 1 |
| P21CDO-S2 | 16 | 64870 | 15 | 13164 | 64662 | 32.75 | 10229 | 3550 | 3 | 6 |
| P21CDO-S4 | 2 | 45304 | 2 | 32314 | 45304 | 33.53 | 32314 | 12990 | 1 | 2 |
| P24CDO-S2 | 6 | 57909 | 4 | 35327 | 57394 | 35.28 | 35327 | 11999 | 1 | 2 |
| P24CDO-S3 | 7 | 95881 | 7 | 32315 | 95881 | 34.87 | 25265 | 12003 | 2 | 4 |
| P24CDO-S4 | 5 | 36414 | 3 | 25786 | 35899 | 36.33 | 25786 | 9589 | 1 | 2 |
| P2CDO3 | 6 | 28449 | 3 | 16508 | 27933 | 36.92 | 16508 | 10776 | 1 | 2 |
| P2CDO4 | 2 | 118775 | 2 | 117324 | 118775 | 32.46 | 117324 | 117324 | 1 | 1 |
| P2CDO-S6 | 12 | 58972 | 9 | 17720 | 58437 | 35.85 | 16508 | 10776 | 2 | 3 |

**Supplementary Table 9**

| **Sample name** | **Bases** | **# CDS** | **# Contigs** | **# Genes** | **# Repeat regions** | **# tmRNA** | **# tRNA** |
| --- | --- | --- | --- | --- | --- | --- | --- |
| 2009-118452 | 16823 | 24 | 3 | 24 | 0 | 0 | 0 |
| 2009-119100 | 33934 | 29 | 10 | 31 | 0 | 0 | 2 |
| 2009-129008 | 91543 | 82 | 7 | 84 | 0 | 0 | 2 |
| 2009-130586 | 45925 | 44 | 5 | 44 | 0 | 0 | 0 |
| 2009-158448 | 11944 | 13 | 1 | 13 | 0 | 0 | 0 |
| 2009-173039 | 98427 | 114 | 10 | 116 | 0 | 0 | 2 |
| 2009-42653 | 76072 | 113 | 2 | 113 | 0 | 0 | 0 |
| 2009-75775 | 103727 | 102 | 5 | 103 | 0 | 0 | 1 |
| 2009-86120 | 30909 | 32 | 3 | 32 | 0 | 0 | 0 |
| 2009-91522 | 100725 | 62 | 128 | 64 | 0 | 0 | 2 |
| 2010-112100-F | 35370 | 35 | 1 | 35 | 0 | 0 | 0 |
| 2010-112100-O | 96410 | 67 | 52 | 69 | 0 | 0 | 2 |
| 2010-112708 | 12861 | 7 | 2 | 7 | 0 | 0 | 0 |
| 2010-112758 | 99371 | 84 | 6 | 86 | 0 | 0 | 2 |
| 2010-112825 | 121971 | 109 | 39 | 126 | 0 | 0 | 17 |
| 2010-113332-F | 81434 | 103 | 3 | 106 | 0 | 0 | 3 |
| 2010-113332-O | 157295 | 124 | 61 | 128 | 0 | 0 | 4 |
| 2010-113862-F | 45139 | 28 | 4 | 30 | 0 | 0 | 2 |
| 2010-113862-O | 19088 | 18 | 8 | 18 | 0 | 0 | 0 |
| 2010-115605-F | 129124 | 109 | 60 | 111 | 0 | 0 | 2 |
| 2010-164712 | 10852 | 13 | 1 | 13 | 0 | 0 | 0 |
| 2010-1718 | 73907 | 74 | 5 | 74 | 0 | 0 | 0 |
| 2010-25654-F | 61396 | 63 | 6 | 63 | 0 | 0 | 0 |
| 2010-25654-O | 46668 | 59 | 3 | 59 | 0 | 0 | 0 |
| 2010-30795 | 31499 | 19 | 3 | 19 | 0 | 0 | 0 |
| 2010-30800 | 13734 | 18 | 3 | 18 | 0 | 0 | 0 |
| 2010-33561 | 123474 | 103 | 38 | 106 | 0 | 0 | 3 |
| 2010-34330 | 44086 | 29 | 8 | 31 | 0 | 0 | 2 |
| 2010-36743 | 39606 | 31 | 5 | 33 | 0 | 0 | 2 |
| 2010-378007-F | 49811 | 50 | 4 | 53 | 0 | 0 | 3 |
| 2010-378007-O | 389850 | 378 | 77 | 385 | 0 | 0 | 7 |
| 2010-43100 | 180604 | 151 | 29 | 159 | 0 | 0 | 8 |
| 2010-8194 | 23265 | 29 | 2 | 29 | 0 | 0 | 0 |
| 2010-88823 | 57782 | 43 | 7 | 43 | 0 | 0 | 0 |
| 2012-164712 | 34899 | 52 | 1 | 52 | 0 | 0 | 0 |
| 2012-191940 | 88273 | 69 | 9 | 69 | 0 | 0 | 0 |
| 2012-37302 | 27257 | 22 | 2 | 22 | 0 | 0 | 0 |
| 2013-101463 | 41717 | 36 | 3 | 38 | 0 | 0 | 2 |
| 2013-39845 | 50780 | 30 | 11 | 30 | 0 | 0 | 0 |
| 2013-42088 | 19757 | 25 | 2 | 25 | 0 | 0 | 0 |
| 2013-87946 | 25812 | 30 | 2 | 30 | 0 | 0 | 0 |
| B124_Slimy-small | 81333 | 74 | 8 | 76 | 0 | 0 | 2 |
| B124_Small-clear | 52718 | 51 | 9 | 51 | 0 | 0 | 0 |
| B124_Small-grey | 96610 | 107 | 33 | 113 | 0 | 0 | 6 |
| B38_Tiny-mucoid | 48049 | 23 | 11 | 26 | 0 | 0 | 3 |
| B124_Slimy-large | 107372 | 80 | 23 | 82 | 0 | 0 | 2 |
| H14O-S1 | 42505 | 36 | 2 | 36 | 0 | 0 | 0 |
| H17O-S1 | 71627 | 68 | 2 | 68 | 0 | 0 | 0 |
| H1O1 | 20198 | 16 | 3 | 16 | 0 | 0 | 0 |
| H21O-S1 | 59772 | 53 | 4 | 53 | 0 | 0 | 0 |
| H21O-S2 | 63765 | 47 | 13 | 47 | 0 | 0 | 0 |
| H21O-S3 | 56635 | 64 | 2 | 64 | 0 | 0 | 0 |
| H21O-S5 | 178260 | 183 | 13 | 191 | 0 | 0 | 8 |
| H22O-S1 | 102760 | 95 | 13 | 97 | 0 | 0 | 2 |
| H23O-S1 | 38998 | 38 | 3 | 38 | 0 | 0 | 0 |
| H9O-S2 | 3970 | 3 | 1 | 3 | 0 | 0 | 0 |
| P13UCO-S3 | 86961 | 93 | 12 | 93 | 0 | 0 | 0 |
| P15UCO-S2 | 47862 | 60 | 4 | 60 | 0 | 0 | 0 |
| P20CDO-S1 | 33544 | 17 | 5 | 19 | 0 | 0 | 2 |
| P20CDO-S2 | 154359 | 183 | 5 | 183 | 0 | 0 | 0 |
| P20CDO-S3 | 166069 | 156 | 13 | 158 | 0 | 0 | 2 |
| P20CDO-S4 | 29033 | 31 | 1 | 31 | 0 | 0 | 0 |
| P21CDO-S2 | 64870 | 56 | 16 | 56 | 0 | 0 | 0 |
| P21CDO-S4 | 45304 | 46 | 2 | 47 | 0 | 0 | 1 |
| P24CDO-S2 | 57909 | 49 | 6 | 55 | 0 | 0 | 6 |
| P24CDO-S3 | 95881 | 76 | 7 | 85 | 0 | 0 | 9 |
| P24CDO-S4 | 36414 | 24 | 5 | 26 | 0 | 0 | 2 |
| P2CDO-S6 | 58972 | 51 | 12 | 53 | 0 | 0 | 2 |
| P2CDO3 | 28449 | 27 | 6 | 29 | 0 | 0 | 2 |
| P2CDO4 | 118775 | 134 | 2 | 134 | 0 | 0 | 0 |

**Supplementary Table 10: Isolates that share plasmid KEGG Orthology (ko0001) KEGG BRITE hierarchies within the different levels.**

|  | 1 | 2 | 3 | 4 | 5 | 6 | 7 | 8 | 9 | 10 | 11 | 12 | 13 | 14 | 15 | 16 | 17 | 18 |
| --- | --- | --- | --- | --- | --- | --- | --- | --- | --- | --- | --- | --- | --- | --- | --- | --- | --- | --- |
| Metabolism | 111 | 36 | 10 | 10 | 3 | 0 | 1 | 0 | 0 | 0 | 0 | 0 | 0 | 0 | 0 | 0 | 0 | 0 |
| Genetic Information Processing | 35 | 5 | 3 | 0 | 1 | 1 | 0 | 0 | 0 | 0 | 1 | 0 | 0 | 0 | 0 | 0 | 0 | 0 |
| Environmental Information Processing | 33 | 6 | 2 | 3 | 3 | 8 | 1 | 0 | 1 | 3 | 0 | 0 | 0 | 0 | 0 | 2 | 1 | 1 |
| Cellular Processes | 23 | 7 | 1 | 1 | 1 | 0 | 0 | 0 | 0 | 0 | 0 | 0 | 3 | 0 | 0 | 1 | 1 | 1 |
| Organismal Systems | 7 | 2 | 0 | 0 | 0 | 0 | 0 | 0 | 0 | 0 | 0 | 0 | 0 | 0 | 0 | 0 | 0 | 0 |
| Human Diseases | 20 | 1 | 1 | 2 | 0 | 2 | 1 | 0 | 0 | 0 | 0 | 0 | 1 | 0 | 0 | 0 | 0 | 0 |

**Supplementary Table 11: Summary statistics for the pangenome of *C. concisus* strains from oral faecal paired participant samples.**

|  |  | **Oral and Faecal** | **Oral** | **Faecal** | **Oral only** | **Faecal only** |
| --- | --- | --- | --- | --- | --- | --- |
| Core genes | (99% <= strains <= 100%) | 646 | 668 | 681 | 0 | 0 |
| Soft core genes | (95% <= strains < 99%) | 0 | 0 | 0 | 0 | 0 |
| Shell genes | (15% <= strains < 95%) | 2824 | 3639 | 3199 | 979 | 552 |
| Cloud genes | (0% <= strains < 15%) | 1389 | 0 | 0 | 0 | 0 |
| Total genes | (0% <= strains <= 100%) | 4859 | 4307 | 3880 | 979 | 552 |
