# Supplementary figures and images for: Comparative genomics of Campylobacter concisus: Analysis of clinical strains reveals genome diversity and pathogenic potential
Source: Emerg Microbes Infect. 2018 Jun 26;7:116. doi: 10.1038/s41426-018-0118-x (PMC6018663; doi:10.1038/s41426-018-0118-x)

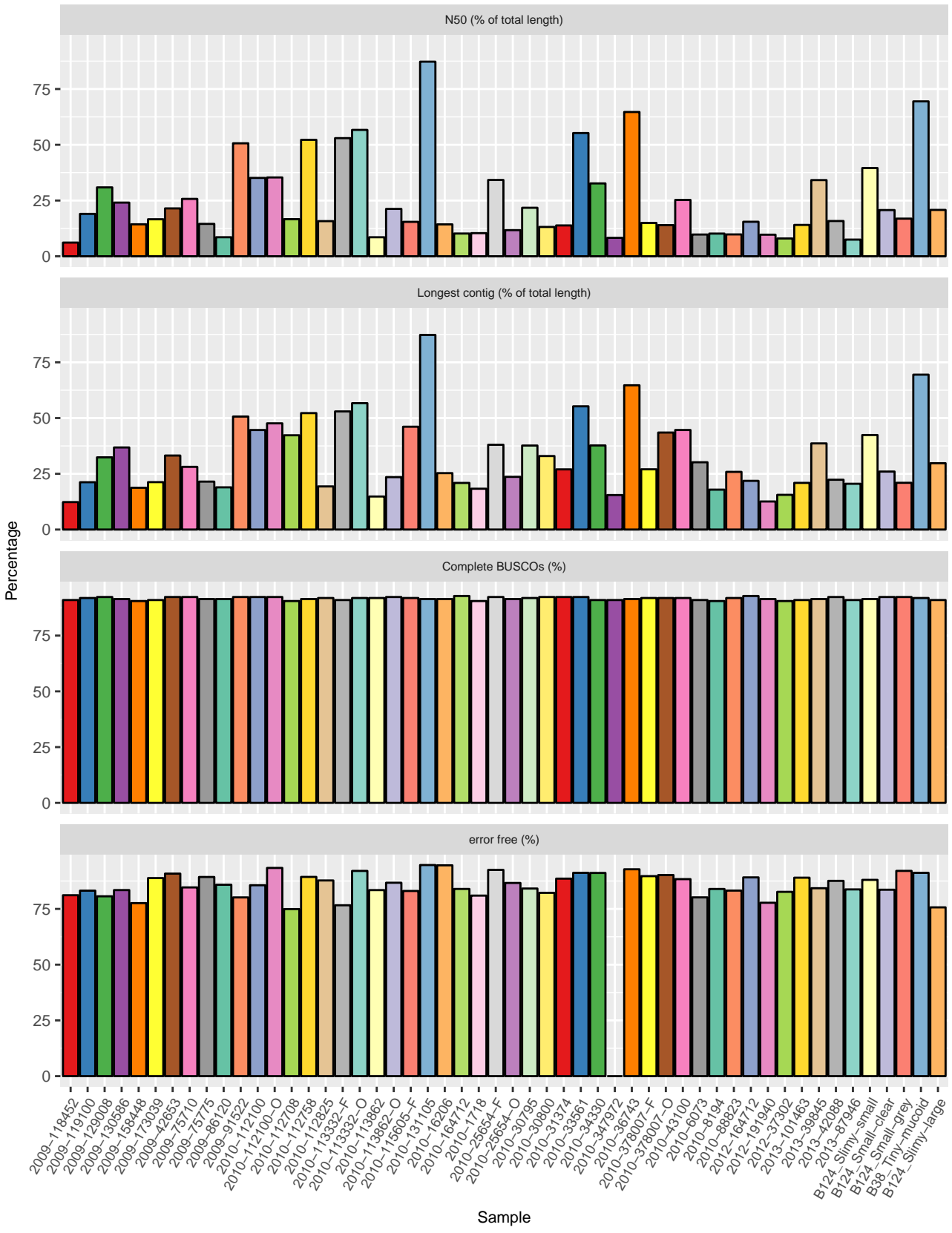

Supplement: Supplementary file 2 — Supplementary Figure 1 [file 41426_2018_118_MOESM2_ESM.pdf]

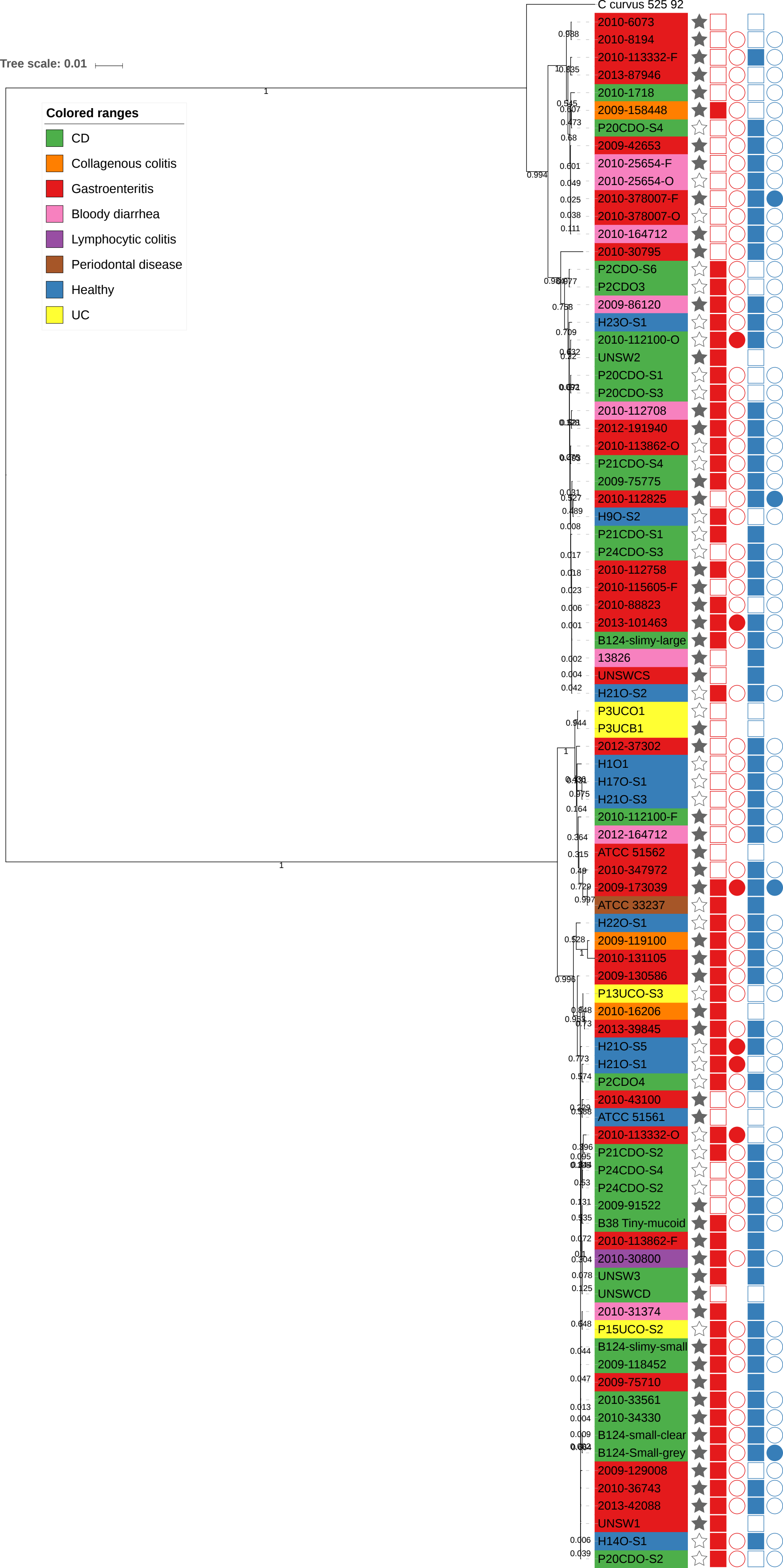

Supplement: Supplementary file 3 — Supplementary Figure 2 [file 41426_2018_118_MOESM3_ESM.pdf]

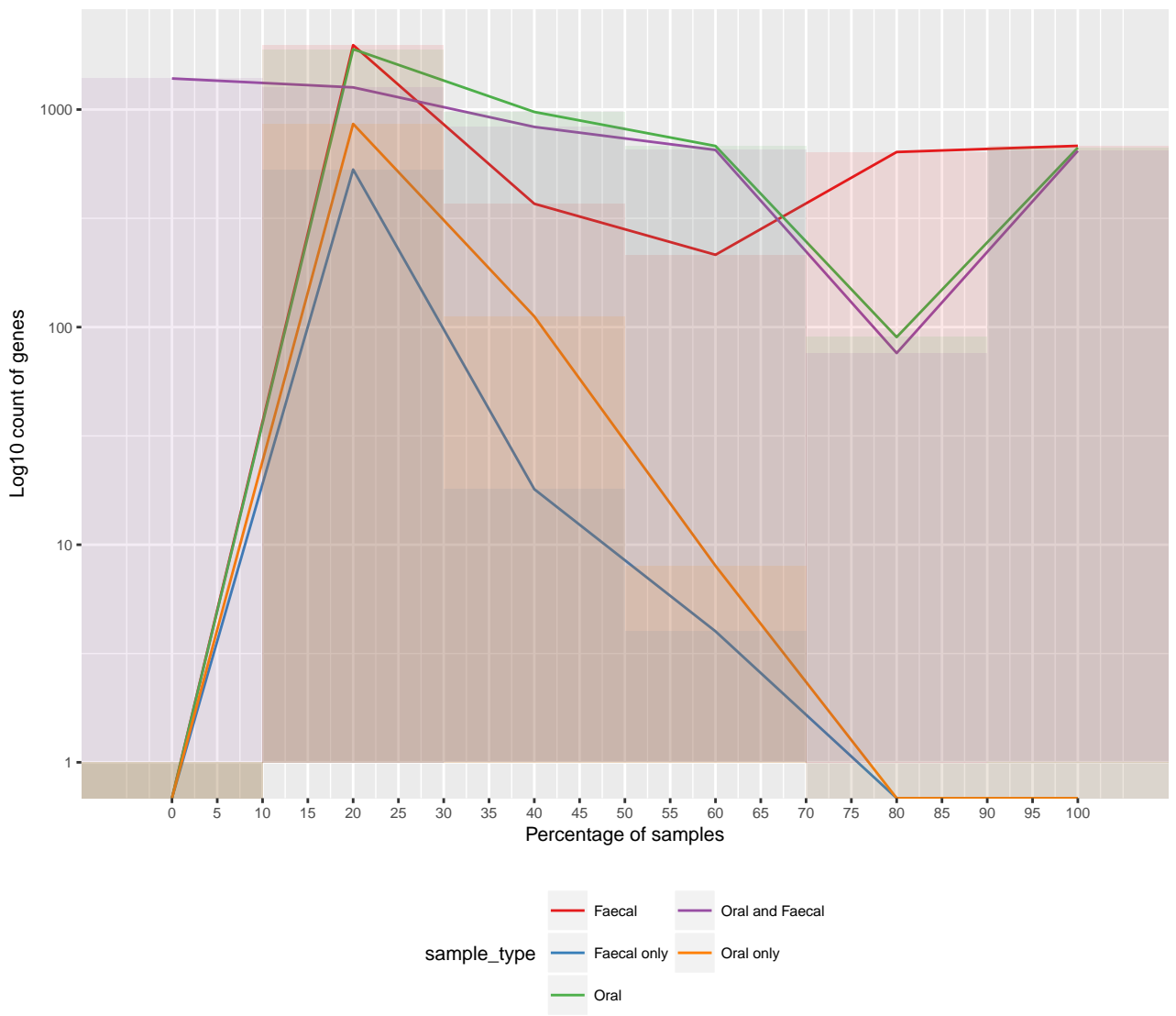

Supplement: Supplementary file 6 — Supplementary Figure 5 [file 41426_2018_118_MOESM6_ESM.pdf]
