# Supplementary material for: Comparative genomics of Campylobacter concisus: Analysis of clinical strains reveals genome diversity and pathogenic potential
Source: Emerg Microbes Infect. 2018 Jun 26;7:116. doi: 10.1038/s41426-018-0118-x (PMC6018663; doi:10.1038/s41426-018-0118-x)
Supplement: Supplementary file 4 — Supplementary Figure 3 [file 41426_2018_118_MOESM4_ESM.pdf]

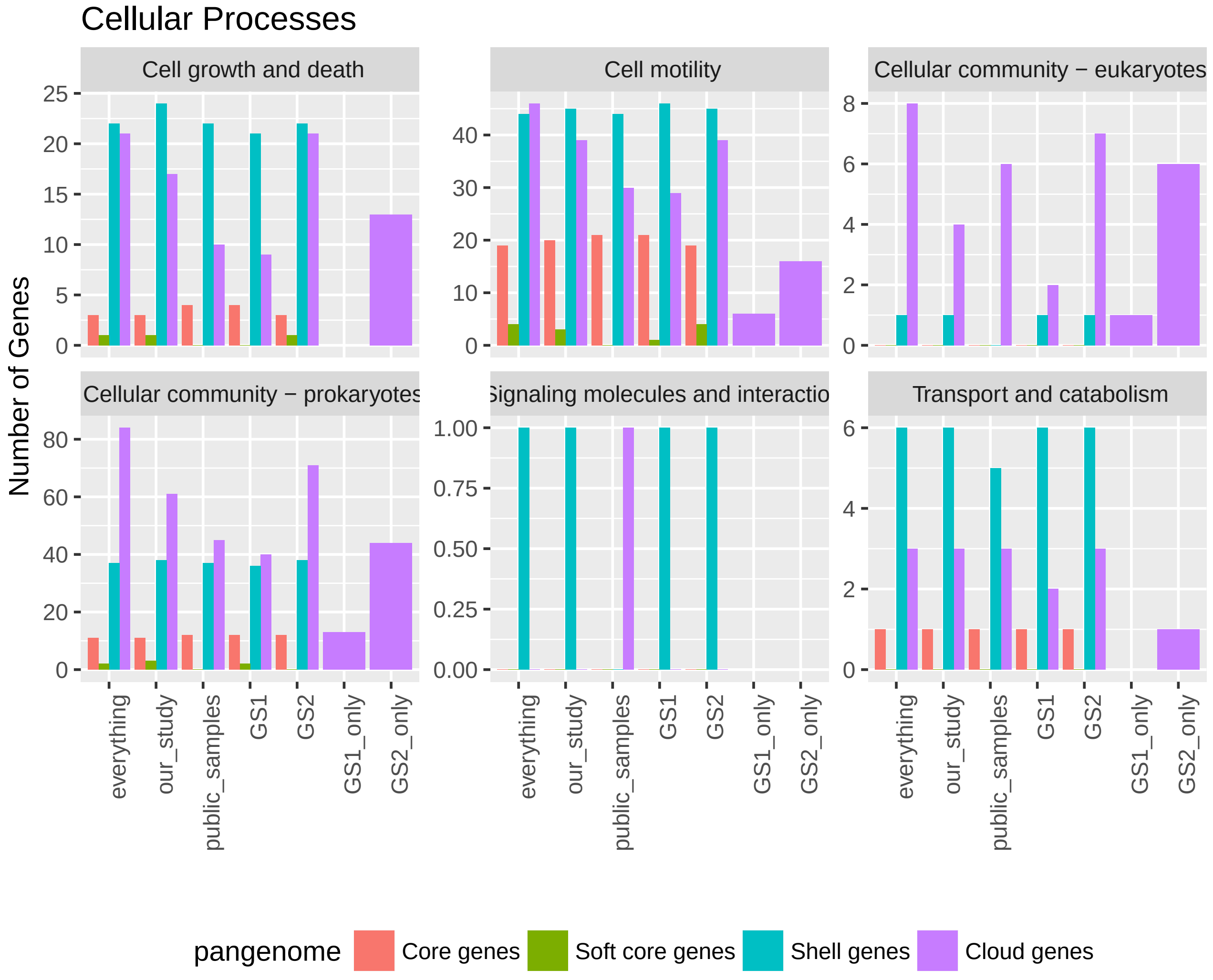

Cellular Processes
Cell growth and death
Cell motility
Cellular community − eukaryotes
Cellular community − prokaryotes
Signaling molecules and interactio
Transport and catabolism
Number of Genes
everything
our_study
public_samples
GS1
GS2
GS1_only
GS2_only
pangenome
Core genes
Soft core genes
Shell genes
Cloud genes

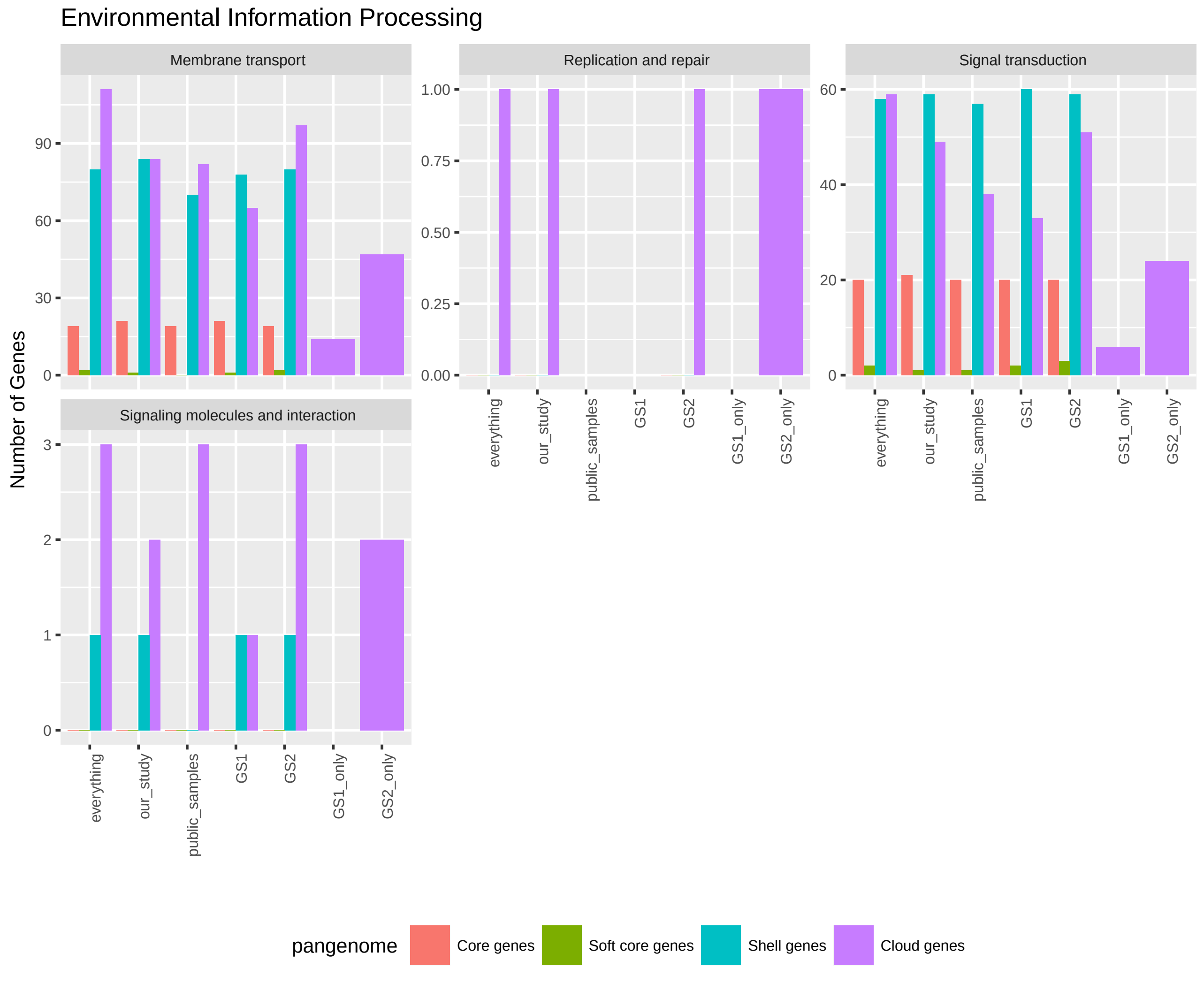

Environmental Information Processing
Membrane transport
Replication and repair
Signal transduction
Signaling molecules and interaction
Number of Genes
0
30
60
90
0.00
0.25
0.50
0.75
1.00
0
20
40
60
0
1
2
3
everything
our_study
public_samples
GS1
GS2
GS1_only
GS2_only
pangenome
Core genes
Soft core genes
Shell genes
Cloud genes

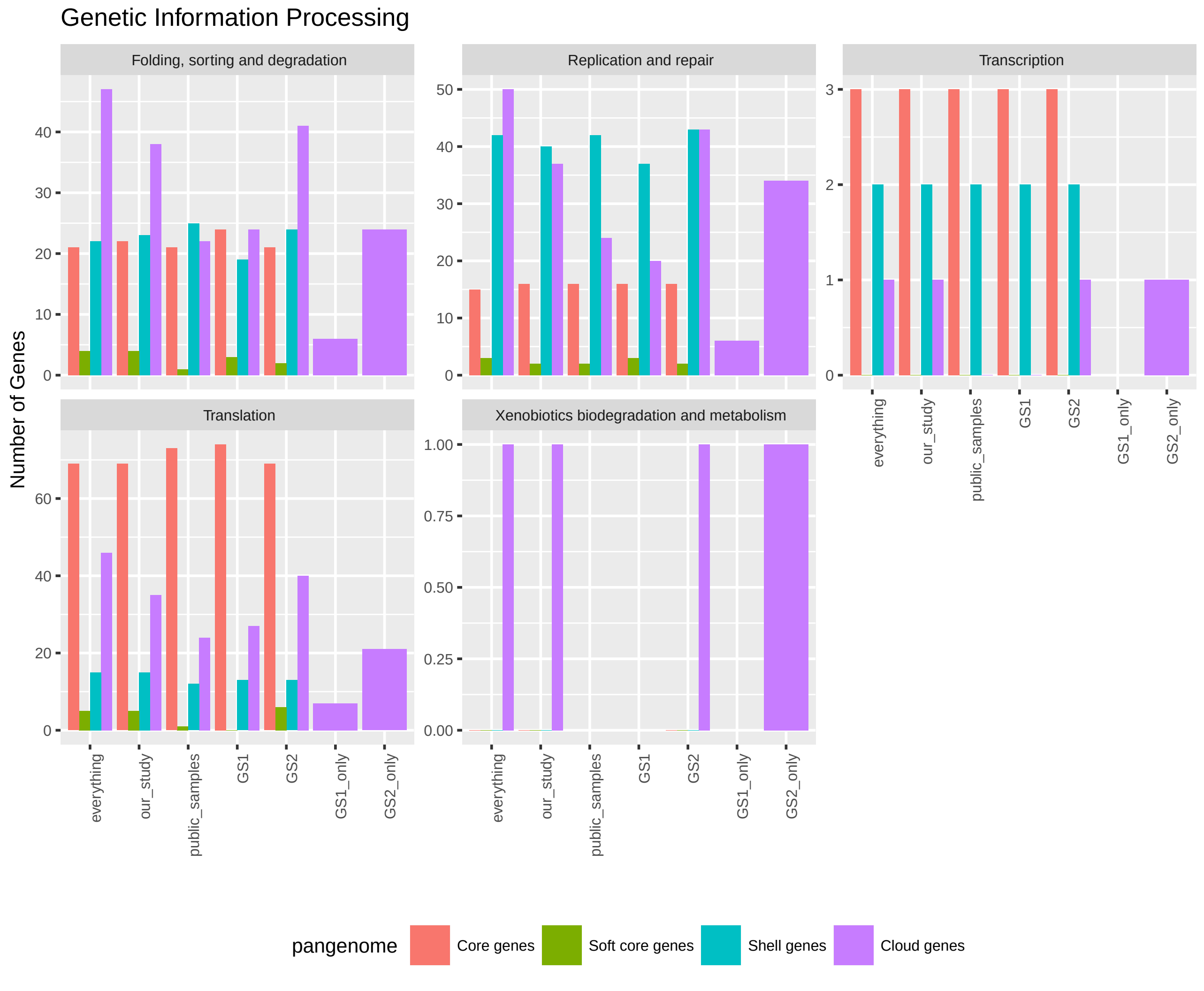
Genetic Information Processing
Folding, sorting and degradation
Replication and repair
Transcription
Translation
Xenobiotics biodegradation and metabolism
Number of Genes
everything
our_study
public_samples
GS1
GS2
GS1_only
GS2_only
pangenome
Core genes
Soft core genes
Shell genes
Cloud genes

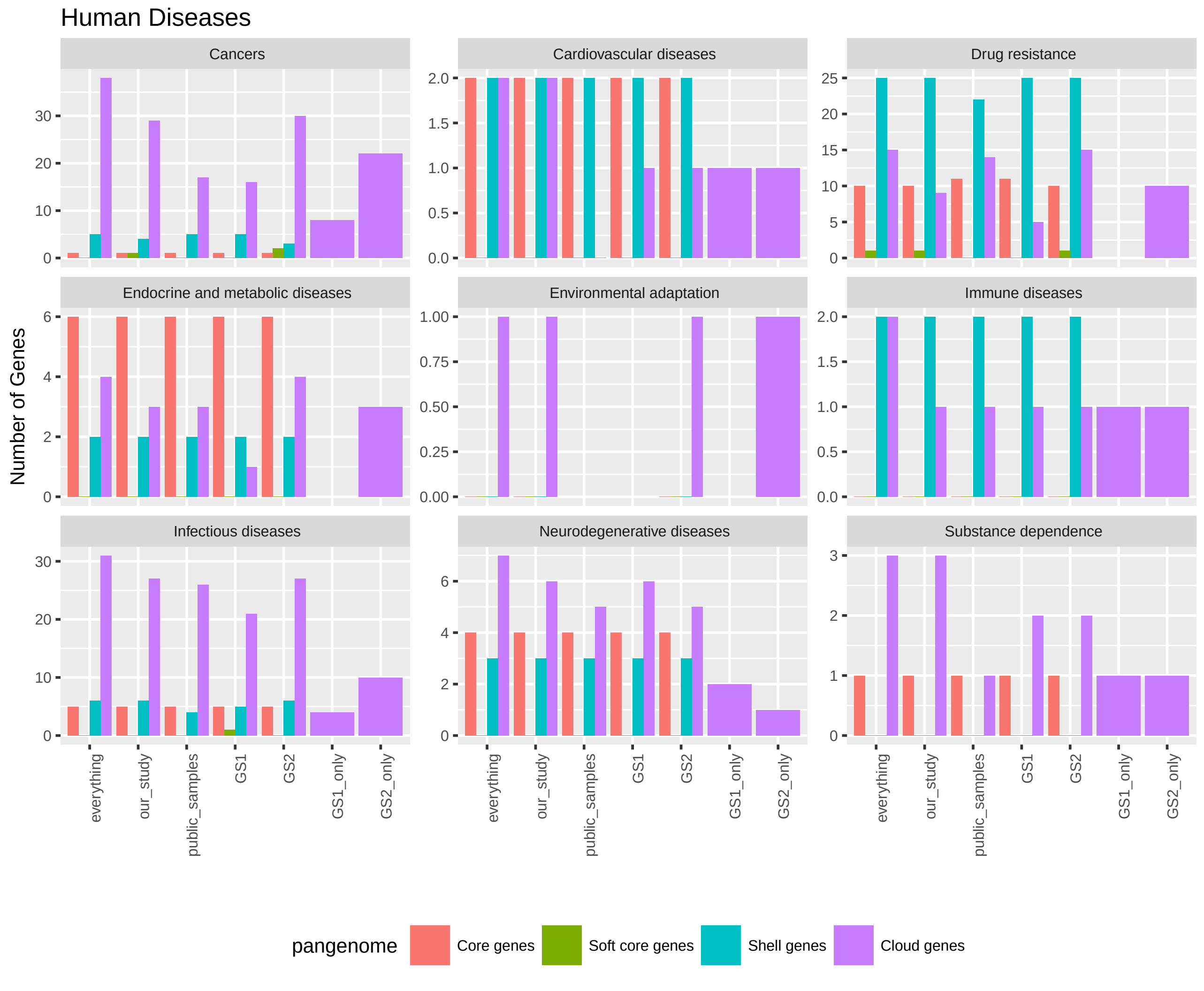

Human Diseases
Cancers
Cardiovascular diseases
Drug resistance
Endocrine and metabolic diseases
Environmental adaptation
Immune diseases
Infectious diseases
Neurodegenerative diseases
Substance dependence
Number of Genes
everything
our_study
public_samples
GS1
GS2
GS1_only
GS2_only
pangenome
Core genes
Soft core genes
Shell genes
Cloud genes

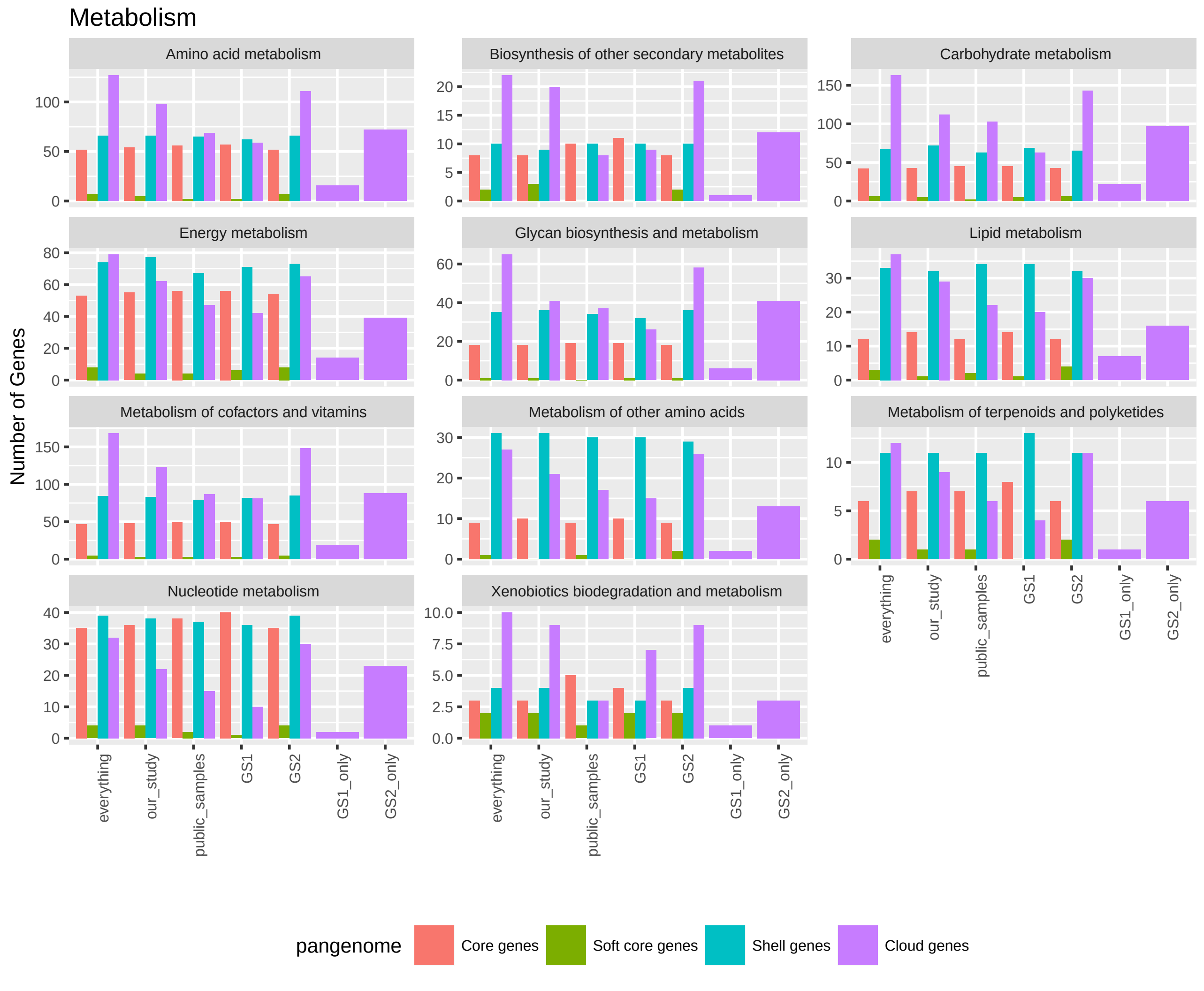

Metabolism
Amino acid metabolism
Biosynthesis of other secondary metabolites
Carbohydrate metabolism
Energy metabolism
Glycan biosynthesis and metabolism
Lipid metabolism
Metabolism of cofactors and vitamins
Metabolism of other amino acids
Metabolism of terpenoids and polyketides
Nucleotide metabolism
Xenobiotics biodegradation and metabolism
Number of Genes
everything
our_study
public_samples
GS1
GS2
GS1_only
GS2_only
pangenome
Core genes
Soft core genes
Shell genes
Cloud genes

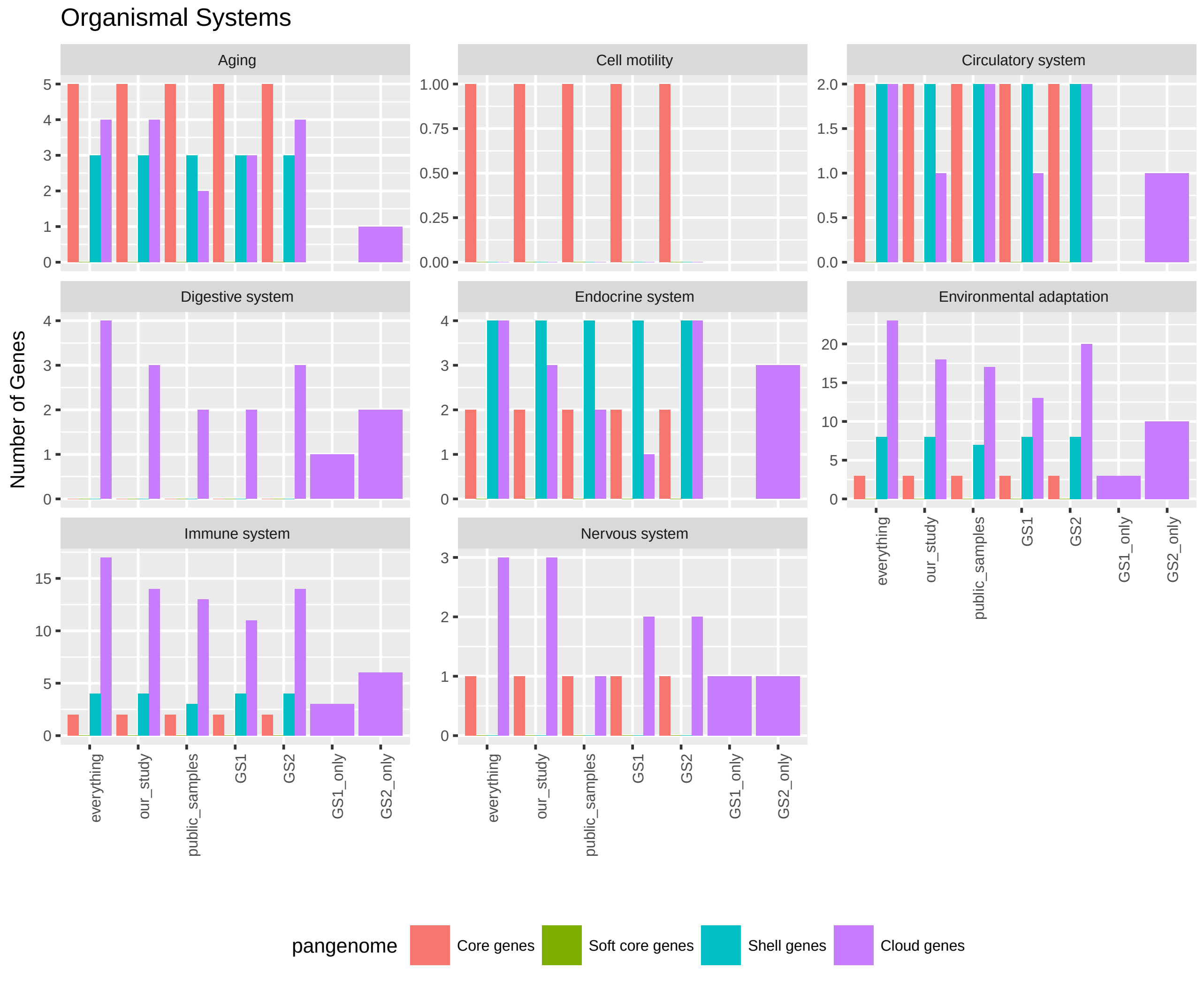

Organismal Systems
Aging
Cell motility
Circulatory system
Digestive system
Endocrine system
Environmental adaptation
Immune system
Nervous system
Number of Genes
everything
our_study
public_samples
GS1
GS2
GS1_only
GS2_only
pangenome
Core genes
Soft core genes
Shell genes
Cloud genes
